# Aβ1‐40 induces venular endothelial cell ferroptosis in cerebral amyloid angiopathy via the ATF3/xCT axis: Insights from single‐cell RNA sequencing

**DOI:** 10.1002/alz.71858

**Published:** 2026-09-20

**Authors:** Chunmei Wu, Xiaodong Ye, Hualin Chen, Yongkang Fang, Yan Lan, Jiahe Ye, Xin Liu, Ruizhi Xiao, Suiqiang Zhu, Shanshan Huang

**Affiliations:** ^1^ Department of Neurology, Tongji Hospital of Tongji Medical College of Huazhong University of Science and Technology; Hubei Key Laboratory of Neural Injury and Functional Reconstruction Huazhong University of Science and Technology Wuhan China; ^2^ Department of Neurology Tongji Hospital of Tongji Medical College of Huazhong University of Science and Technology Wuhan China; ^3^ Department of Geriatrics Tongji Hospital of Tongji Medical College of Huazhong University of Science and Technology Wuhan China

**Keywords:** activating transcription factor 3, cerebral amyloid angiopathy, ferroptosis, single‐cell RNA sequencing, venular

## Abstract

**INTRODUCTION:**

Amyloid‐beta (Aβ)‐induced microvascular injury is a key pathological feature of cerebral amyloid angiopathy (CAA). Recent evidence suggests that ferroptosis is implicated in Aβ pathology; however, its cell‐type specificity and regulatory mechanisms within the cerebral microvasculature in CAA remain unclear.

**METHODS:**

Single‐cell RNA sequencing (scRNA‐seq) was performed on cortical microvessels from 11‐month‐old APP23 mice and wild‐type littermates to identify cell‐type‐specific ferroptosis signatures. Biochemical, histopathological, and intervention experiments were further conducted to validate the findings and investigate the underlying mechanisms.

**RESULTS:**

Venous endothelial cluster exhibited the most prominent enrichment of ferroptosis signatures in scRNA‐seq analysis, with subsequent experiments demonstrating progressive ferroptosis‐related alterations in cortical venular endothelial cells (vECs) during CAA development. Mechanistically, Aβ1‐40 increased activating transcription factor 3 (ATF3) expression and its occupancy at the *Slc7a11* promoter, whereas ATF3 silencing restored *Slc7a11*/xCT expression, supporting ATF3‐mediated *Slc7a11/*xCT repression and consequent impairment of glutathione‐dependent antioxidant defense. Importantly, endothelial ATF3 knockdown attenuated ferroptosis‐related alterations in vECs, reduced cerebral Aβ burden, and ameliorated cognitive deficits in APP23 mice.

**DISCUSSION:**

This study uncovers a previously underrecognized Aβ‐driven microvascular injury characterized by vEC ferroptosis and highlights the ATF3/xCT axis as a potential therapeutic target in the early stage of CAA.

## Highlights

- Single‐cell RNA‐seq of cortical microvessels identifies a prominent ferroptosis signature within the venous endothelial cluster in cerebral amyloid angiopathy (CAA).
- Amyloid‐β1‐40 (Aβ1‐40) induces ferroptosis in venular endothelial cells (vECs) through the ATF3/xCT axis.
- Endothelial activating transcription factor 3 (ATF3) knockdown attenuates vEC ferroptosis, reduces cerebral Aβ burden, and ameliorates cognitive deficits in APP23 mice.

## 1 BACKGROUND

Cerebral amyloid angiopathy (CAA) is a type of cerebral small vessel disease (CSVD) characterized by amyloid‐β (Aβ) deposition in the media and adventitia of cortical and leptomeningeal small vessels.^1^ As a major cause of spontaneous lobar intracerebral hemorrhage and progressive cognitive decline in the elderly,^2^ CAA involves intricate pathophysiological mechanisms that are not fully understood, consequently leading to a lack of effective targeted therapies.

Aβ homeostasis depends on a dynamic balance between its production and clearance, and impaired clearance is recognized as a major factor in pathological Aβ accumulation.^3^ Emerging evidence suggests that the venous system contributes to Aβ clearance. As part of the glymphatic system, perivenous spaces serve as important routes for removing metabolic waste from the brain.^4^ Moreover, patrolling monocytes preferentially target Aβ‐positive veins, where they capture and eliminate vascular Aβ.^5^ Venous dysfunction may therefore impair Aβ clearance and promote vascular or parenchymal amyloid deposition, a possibility supported by neuroimaging evidence of an association between abnormal cerebral venous drainage and greater cerebral Aβ burden.^6^ Additional clinical observations support a broader role for the venous system in human Aβ‐related disorders. In Alzheimer's disease (AD), venous collagenosis has been associated with white matter hyperintensities,^7^ linking venous pathology to white matter injury in the context of Aβ‐related neurodegeneration. In CAA, the spatial connection between small veins and cerebral microbleeds (CMBs) revealed by high‐field magnetic resonance imaging (MRI) further implicates the venous involvement in amyloid‐mediated hemorrhagic lesions.^8^ Nevertheless, compared with the arterial circulation, the venous side remains largely underexplored in CAA, highlighting the need for a more comprehensive understanding of this disease across the entire arteriovenous spectrum.

Ferroptosis, an iron‐dependent form of programmed cell death (PCD) driven by lipid peroxidation,^9^ participates in the pathogenesis of several central nervous system disorders including neurodegenerative,^10^ cerebrovascular,^10^ and epileptic conditions.^11^ Notably, ferroptosis is implicated in Aβ pathology through bidirectional regulatory mechanisms. Iron as a key mediator of ferroptosis enhances Aβ toxicity^12^ and promotes its aggregation,^13^ while Aβ facilitates the reduction of Fe^3^^+^ to Fe^2^^+^ thereby amplifying Fenton reaction‐mediated oxidative damage.^14^ Building on this mechanistic link, cerebral vascular iron deposition has been reported in CAA.^15^ In addition, substantial evidence further suggests ferroptosis as a critical mechanism in AD,^16^, ^17^ wherein Aβ triggers ferroptosis across various cell types such as neurons^18^ and pericytes.^19^ CAA is considered a sister disease of AD, as both share the common pathological hallmark of Aβ deposition and frequently co‐occur.^20^ However, it remains unclear whether a similar ferroptotic mechanism operates in cerebral small vessel cells in CAA, where the predominant Aβ species (Aβ1‑40) accumulates in the vascular walls rather than in the brain parenchyma.^20^

**RESEARCH IN CONTEXT**

1. **Systematic review**: The authors searched the literature using established databases (e.g., PubMed). Previous studies have implicated ferroptosis in amyloid‐β (Aβ) ‐related pathology, whereas its involvement in the cerebral microvasculature of cerebral amyloid angiopathy (CAA) remains poorly characterized, particularly at single‐cell resolution. Relevant studies are appropriately cited.
2. **Interpretation**: Combining single‐cell profiling of cortical microvessels with pathological and mechanistic studies, we show that ferroptosis in venular endothelial cells (vECs) is an underrecognized feature of early CAA, with Aβ1‐40 promoting this process through the ATF3/xCT axis and endothelial activating transcription factor 3 (ATF3) knockdown reducing cerebral Aβ burden and ameliorating cognitive deficits.
3. **Future directions**: Future studies should focus on: (a) elucidating how vEC ferroptosis affects venular function and cerebral Aβ clearance; (b) developing gene delivery vectors with greater specificity for brain vECs; and (c) establishing the clinical relevance of findings from mouse models through human studies.

A major challenge in studying the cerebral small vasculature is isolating it from the surrounding parenchyma. Microvessel extraction techniques^21^, ^22^ offer a solution. Recent bulk proteomic studies on cerebrovasculature have identified several proteins or pathways linked to CAA pathophysiology.^23^, ^24^ However, the cerebrovascular system forms a complex hierarchical network^25^ whose architecture extends to a remarkably diverse microscopic makeup, including heterogeneous populations of endothelial, mural, fibroblast, and immune cells.^26^ Such cellular heterogeneity limits the resolution of bulk omics in deciphering cell‐type–specific functional alterations. To address this limitation, the integration of microvessel extraction techniques with single‐cell RNA sequencing (scRNA‐seq)^27^ enables the mapping and functional profiling, including the analysis of ferroptosis, across diverse cerebrovascular cell types in CAA.

In this study, we used scRNA‐seq to conduct transcriptomic analysis of the cortical microvasculature in APP23 mice, a well‐established mouse model of CAA. Our analysis revealed ferroptosis enrichment across multiple endothelial and mural cell types, with the highest enrichment score observed in venous endothelial cluster. Integrated in vivo and in vitro experiments further demonstrated that Aβ1‐40 promotes ferroptosis in venular endothelial cells (vECs) via activation of the ATF3/xCT pathway. Importantly, endothelial activating transcription factor 3 (ATF3) knockdown significantly reduced cerebral Aβ deposition and improved cognitive function in APP23 mice. These findings provide novel insights into CAA pathogenesis and may support the development of targeted therapeutic strategies.

## 2 METHODS

### 2.1 Experimental animals

Male heterozygous APP23 transgenic mice (APP23) aged 6 to 24 months were used as a murine model of CAA,^28^ with age‐matched male wild‐type (WT) littermates employed as controls. The APP23 mice (B6.Cg‐Tg(Thy1‐APP)3Somm/J, RRID:IMSR_JAX:030504) were acquired from The Jackson Laboratory (Bar Harbor, ME, USA). All mice were housed in the specific pathogen‐free (SPF) grade conditions at 22 ± 1°C with a 12‐h light/dark cycle and had ad libitum access to food and water. Mouse genotypes were determined by polymerase chain reaction (PCR) analysis of tail biopsy DNA using a commercial kit (PD101‐01, Vazyme). Animal experiments were approved by the Tongji Medical College Animal Care and Use Committee (Huazhong University of Science and Technology; Ethics Approval No. TJH‐202410007).

### 2.2 Tissue preparation

Mice were deeply anesthetized with isoflurane. A thoracotomy was performed, followed by transcardial perfusion with ice‐cold phosphate‐buffered saline (PBS). For immunofluorescence staining, perfusion with pre‐chilled 4% paraformaldehyde (PFA) was subsequently conducted. The brains were then rapidly dissected, post‐fixed in 4% PFA overnight at 4°C, and dehydrated through a graded series of sucrose solutions (15%, 20%, and 30% [w/v]). After dehydration, the brains were snap‐frozen, and coronal sections were cut at a thickness of 12 µm using a cryostat (CryoStar NX50, Thermo Scientific, USA). The brain sections were stored at −80°C. For molecular analyses, the cerebral cortex was carefully dissected after PBS perfusion, snap‐frozen in liquid nitrogen, and stored at −80°C.

Paraffin‐embedded brain sections from postmortem sporadic CAA patients and controls were provided by the National Human Brain Bank for Development and Function, Chinese Academy of Medical Sciences and Peking Union Medical College, Beijing, China. The demographic and clinical characteristics of the patients and controls are summarized in Table S1.

### 2.3 Aβ oligomer preparation

The Aβ1‐40 oligomers were prepared as follows. First, synthetic Aβ1‐40 peptide (AMYD‐009, Chinesepeptide) was dissolved in hexafluoroisopropanol (HFIP) to yield a 1 mM stock solution. This solution was aliquoted and evaporated under a fume hood to form peptide films, which were stored at −20°C. For oligomerization, a peptide film was reconstituted in dimethyl sulfoxide (DMSO) to obtain a 5 mM solution, followed by dilution with sterile PBS to a final concentration of 100 µM. This solution was incubated at 4°C for 24 h to facilitate oligomer formation. The resulting oligomers were stored at −80°C for short‐term use. Prior to experiments, oligomers were diluted in culture medium to a working concentration.

### 2.4 Cortical microvessel purification and Aβ1‐40 treatment

Purification of cortical microvascular fragments from mice were adapted from previous study.^21^ For each mouse, the isolated cerebral cortex was homogenized in 5 mL ice‐cold B1 buffer (1 × Hanks’ buffered saline solution [HBSS] containing 10 mM HEPES) using a 7 mL glass homogenizer (15 strokes of a loose‐fitting pestle and 15 strokes with a tight‐fitting pestle). After centrifuged at 2000 × *g* for 10 min at 4°C, the pellet was resuspended in 5 mL B2 solution (18% Dextran‐70 (D8821, Sigma‐Aldrich) in B1 buffer), mixed thoroughly, and centrifuged at 4400 × *g* for 15 min at 4°C. The upper myelin layer was resuspended in B2 solution, and the centrifugation step was repeated. The microvessel pellet was resuspended in 1 mL B3 solution (B1 buffer supplemented with 1% bovine serum albumin [BSA]), and the suspension was filtered through a 20 µm mesh filter. After washing by B3 solution, the filter was inverted over a new 50 mL tube, and the captured microvessels were flushed through with 25 mL B3 solution. Following centrifugation (2000 × *g*, 10 min, 4°C), the pelleted microvessels were either processed immediately for downstream applications—such as immunostaining, scRNA‐seq, or Aβ stimulation assays—or snap‐frozen and stored at −80°C for subsequent biomolecular analysis (e.g., protein extraction or analysis with commercial kits).

For Aβ1‐40 stimulation experiments, the microvessels were isolated under sterile conditions. The freshly isolated microvessels were then treated with 1 µM oligomeric Aβ1‐40 or vehicle (0.02% DMSO) for 6 h.

### 2.5 Liproxstatin‐1 treatment in vivo

APP23 mice aged 10‐month received intraperitoneal injections of Liproxstatin‐1 (Lip‐1, 10 mg/kg; Merck, SML1414) or vehicle control (1.5% DMSO in 0.9% saline) once daily for 21 consecutive days.

### 2.6 scRNA‐seq

#### 2.6.1 Preparation of single‐cell suspension

Fresh microvessel pellets were immediately resuspended in tissue preservation solution provided by Beijing SeekGene Biotech Co., Ltd., and the samples were shipped on ice to their facility for further processing. Single‐cell suspensions were prepared and subjected to sequencing after passing quality control. To meet the required cell load for sequencing, cortical microvessels from two mice were pooled into one sample. A total of three samples each for APP23 and WT mice (six 11‐month‐old mice per genotype) were submitted for analysis. Libraries were prepared using SeekOne^®^ Digital Droplet Single Cell 3' library preparation kit (SeekGene, Catalog No. K00202). Briefly, an appropriate number of cells were mixed with reverse transcription reagent and then added to the sample well in the SeekOne^®^ chip S3. Subsequently, Barcoded Hydrogel Beads and partitioning oil were dispensed into corresponding wells separately in chip S3. After emulsion droplet generation, reverse transcription was performed at 42°C for 90 min and inactivated at 85°C for 5 min. Next, cDNA was purified from the emulsion droplets and amplified in a PCR reaction. The amplified cDNA product was then cleaned, fragmented, end‐repaired, A‐tailed, and ligated to a sequencing adaptor. Finally, the indexed PCR was performed to amplify the DNA representing 3' polyA region of expressed genes, which also contained the Cell Barcode and Unique Molecular Index. The indexed sequencing libraries were cleaned up with VAHTS DNA Clean Beads (Vazyme, N411‐01), analyzed by Qubit (Thermo Fisher Scientific, Q33226), and Bio‐Fragment Analyzer (Bioptic, Qsep400). The libraries were then sequenced on Illumina NovaSeq 6000 with PE150 read length. This sequencing process was carried out by Beijing SeekGene BioSciences Co. Ltd.

#### 2.6.2 scRNA‐seq data analysis

The SeekSoulTools (v. 1.2.2) was applied to align reads for each sample based on the mouse reference genome GRCm38/mm10. Seurat R package (v. 4.4.0) was used for downstream analyses. Further quality control was applied to cells based on these thresholds: (1) the number of expressed genes larger than 200; (2) the unique molecule identifier (UMI) counts per cell larger than 2000; and (3) the cells with less than 5% mitochondrial RNA content. DoubletFinder R package was applied to remove potential doublets. The filtered gene expression matrix for each sample was normalized and scaled by “NormalizeData” and “ScaleData” functions in Seurat. Harmony R package was used to adjust batch effects between different samples and integrate the gene expression matrices of all samples. Finally, we identified 32,325 genes and detected 25,919 cells from six samples. We performed principal component analysis (PCA) on the corrected expression matrix using highly variable genes (HVGs) identified by “FindVariableGenes” function. Next, “RunPCA” function was used to perform the PCA and “FindNeighbors” function was used to construct a K‐nearest‐neighbor graph. The most representative principal components were used to determine different cell types with “FindCluster” function with the resolution set as default of 0.8. We annotated cell types, and six clusters were identified based on expression of the following marker genes. For example, Pecam1 for endothelial cells, Myh11 for mural cells, S100a8 for monocytes, C1qb for microglia, Cd3e and Ms4a1 for NK/T/B cells, and Alas2 for erythrocytes.

#### 2.6.3 Identification of subpopulations of endothelial and mural cells

Three endothelial cell subtypes were identified under the resolution of 0.4 and annotated based on expression of some markers, including arteries (Bmx, Cytl1, Eln, Fbln5, Mgp, Vegfc, Alpl, Efnb2, Gkn3, Hey1, and Sema3g), capillaries (Cxcl12, Mfsd2a, and Rgcc), and venules (Slc16a1, Ackr1, Apod, Fth1, Lcn2, Nr2f2, Slc38a5, Icam1, Vcam1, and Lrg1). Three mural cell subtypes were identified under the resolution of 0.4 and annotated based on expression of some markers, including arterial smooth muscle cells (Acta2, Tagln, Cnn1, and Myh11), venous smooth muscle cells (S1pr3, Trpv2, Jph2, and Atp2b4), and pericytes (Vtn, Higd1b, Pdgfrb, and Cox4i2).

#### 2.6.4 Differential expression analysis

To identify differentially expressed genes (DEGs) for each cell subtype, the functions “FindAllMarkers” (multiple condition comparisons) and “FindMarkers” (two condition comparisons) of the Seurat package were used with default parameters. The expression differences with *p* < 0.05 and log2(fold change [FC]) > 0.25 were considered as DEGs.

#### 2.6.5 Function enrichment and gene set enrichment analysis

The Kyoto Encyclopedia of Genes and Genomes (KEGG) enrichment analysis for DEGs of subpopulations were performed using the clusterProfiler R package (version 4.7.1.2). Pathways with an adjusted *p*‐value of less than 0.05 were considered significantly enriched by DEGs. Gene set enrichment analysis (GSEA) was performed to evaluate the ferroptosis activity of venous ECs between two conditions. The ferroptosis gene set was procured from the MSigDB (M39768, https://www.gsea‐msigdb.org/gsea/msigdb/cards/WP_FERROPTOSIS.html) and translated into their mouse homologs using homologene R package.

#### 2.6.6 Gene module enrichment analysis

Five PCD gene sets including apoptosis, autophagy, ferroptosis, necroptosis, and pyroptosis were obtained from the study by Liu et al.^29^ The scores of these gene sets in indicated subtypes were evaluated by UCell R package, AUCell R package, and “AddModuleScore” function of Seurat R package and compared between two conditions.

In addition, we applied irGSEA R package to determine the activities of each PCD in subtypes of endothelial and mural cells. Six implemented methods including AUCell, UCell, singscore, ssGSEA, JASMINE, and Viper were used in this analysis with other default settings.

#### 2.6.7 Protein–protein interaction

A protein–protein interaction (PPI) network was constructed using differentially expressed ferroptosis‐related genes identified in vECs from APP23 versus WT mice. The STRING database was employed to generate the PPI network, which was subsequently imported into Cytoscape software (v.3.10.3) for further analysis. Hub genes within the network were identified using the CytoHubba plugin. Based on the degree algorithm, the gene with the highest degree of connectivity was defined as the hub gene.

### 2.7 Immunofluorescence staining

Frozen brain slices were rewarmed at room temperature for 20 min. Following three 5 min PBS washes, the slices were permeabilized with immunostaining permeabilization buffer (P0096, Beyotime) for 10 min and subsequently blocked with immunostaining blocking buffer (P0260, Beyotime) for 20 min at room temperature. Then, the slices were incubated overnight at 4°C with primary antibodies. Following the primary antibody incubation, the slices were washed three times with PBS for 5 min each and incubated with corresponding fluorescently‐labeled secondary antibodies for 1 h at room temperature, protected from light. After three additional 5 min washes with PBS in the dark, brain slices were air‐dried and mounted using an anti‐fade mounting medium containing 4,6‐diamidino‐2‐phenylindole (DAPI). The same staining procedure was used for pre‐fixed cultured cells and isolated microvessels. Paraffin sections from CAA patients and controls underwent standard dewaxing, rehydration, and antigen retrieval before being stained following the same protocol as for frozen sections. Finally, images were acquired using a fluorescent microscope (BX51, Olympus, Japan) or confocal microscopy (FV1200, Olympus, Japan) and analyzed with ImageJ software. To ensure comparability, fixed intensity and voxel thresholds were applied identically across all samples. The primary antibodies used for immunofluorescence staining were as follows: 6E10 (1:500, 803001, Biolegend), rat anti‐CD31 (1:100, 550274, R&D Pharmingen), rabbit anti‐alpha‐smooth muscle actin (a‐SMA) (1:500, 19245S, Cell Signaling Technology), mouse anti‐a‐SMA (1:200, sc‐53142, Santa cruz biotechnology), rabbit anti‐glutathione peroxidase 4 (GPX4) (1:200, ab125066, abcam), rabbit anti‐cystine/glutamate Antiporter (system Xc^−^)(xCT) (1:200, A2413, ABclonal), rabbit anti‐ferroportin (FPN) (1:200, NBP1‐21502, Nocus Biologicals), and rabbit anti‐ATF3 (1:100, EPR19488, Abcam).

### 2.8 Enhanced Perls' Prussian blue staining

The enhanced Perls’ Prussian Blue staining was conducted according to the manufacturer's protocol (G1428, Solarbio). Briefly, rewarmed slices were incubated with Perls working solution at 37°C for 20 min. After washed by deionized water, slices were treated with the kit's incubation solution (37°C, 20 min), followed by PBS washes. The signal was enhanced by incubation with the enhancement solution (37°C, 15 min). Following a counterstain and a quick rinse, slices were dehydrated through a graded ethanol series, cleared in xylene, and mounted with neutral balsam. Images were acquired using a bright‐field microscope.

### 2.9 Congo red staining

Congo red staining was performed using a commercial assay kit (G1530; Solarbio) according to the manufacturer's instructions. Briefly, sections were stained with Bennhold's hematoxylin for 5 min, differentiated in acid differentiation solution for 2–5 s, and blued in Scott's solution 3 min, with distilled water rinses between steps. Congo red staining was then conducted at room temperature for 30 min, followed by brief differentiation (2–3 s) and a tap water wash. Finally, sections were dehydrated, cleared in xylene, and mounted. Images were acquired using a bright‐field microscope.

### 2.10 MCT1 co‐staining with enhanced Perls’ Prussian blue or Congo red

In the immunohistochemistry stage, frozen sections from mice were rewarmed (20 min, room temperature) and underwent antigen retrieval with ethylenediaminetetraacetic acid (EDTA) (10009717, Sinopharm; 10 min, room temperature) followed by pepsin treatment (Biossci; 15 min, 37°C). After blocking with 10% goat serum (30 min, room temperature), sections were incubated overnight at 4°C with rabbit anti‐Monocarboxylate Transporter 1 (MCT1) (1:200, 20139‐1‐AP, Proteintech). The next day, sections were incubated with a goat anti‐rabbit secondary antibody (45 min, 37°C) and developed with AEC (AEC‐0037, MXB) or DAB (DAB‐4033, MXB) substrate (20 min), as appropriate. The reaction was stopped by rinsing with water. Paraffin sections of sporadic CAA and control cases underwent standard dewaxing and rehydration, followed by EDTA microwave‐assisted antigen retrieval. After cooling, sections were rinsed in PBS (3 min) and treated with 3% H_2_O_2_ (30 min, room temperature) for endogenous peroxidase blockade. The remaining steps matched the protocol for frozen sections.

After immunohistochemistry, slices were further processed for enhanced Perls’ Prussian blue or Congo red staining. The sections were finally dehydrated, cleared in xylene, and mounted. Images were acquired using a bright‐field microscope.

### 2.11 Western blot

To extract total protein, samples were homogenized RIPA lysis buffer containing protease inhibitors (AR0102S, Boster). Protein concentration was determined using a BCA assay kit (KTD3010, Abbkine). Extracts (20–40 µg protein) separated by 10% sodium dodecyl sulfate‐polyacrylamide gel electrophoresis (SDS‐PAGE) and transferred to 0.22 µm polyvinylidene fluoride (PVDF) membranes. The membranes were blocked with 5% non‐fat milk for 1 h at room temperature and subsequently incubated overnight at 4°C with the following primary antibodies: β‐actin (1:4000, GB15003, Servicebio), 6E10 (1:1000, 803001, BioLegend), anti‐FTH (1:500, sc‐376594, Santa Cruz Biotechnology), anti‐FTL (1:2000, 10727‐1‐AP, Proteintech), anti‐Ferritin (1:2000, ab75973, Abcam), anti‐Ferroportin (1:1000, NBP1‐21502, Nocus Biologicals), anti‐TFRC (1:500, sc‐65882, Santa Cruz Biotechnology), anti‐DMT1 (1:2000, 20507‐1‐AP, Proteintech), anti‐GPX4 (1:2000, ab125066, Abcam), anti‐xCT (1:1000, A2413, Abclonal), anti‐ACSL4 (1:5000, 22401‐1‐AP, Proteintech), anti‐ATF3 (1:1000, EPR19488, Abcam). After washing with TBST, the membranes were incubated with corresponding horseradish peroxidase (HRP)‐conjugated secondary antibodies for 1 h at room temperature. Immunoreactive bands were visualized with a chemiluminescence kit (G2020, Servicebio) and the Gelview 6000 pro imaging system (BLT, China). Quantitative analysis was performed using ImageJ software (version 1.53). Protein expression levels were normalized to β‐actin.

### 2.12 RNA extraction and real‐time quantitative PCR

Total RNA was isolated using the SteadyPure RNA Extraction Kit (AG21024, Accurate Biology). Subsequently, 1 µg of RNA was reverse‐transcribed into cDNA with the PrimeScript RT Master Mix (RR036A, TAKARA). For quantitative PCR (qPCR), reactions were set up in a 10 µL volume with SYBR Green Premix Pro Taq HS qPCR Kit (AG11701, Accurate Biology) and run on a Bio‐Rad CFX96 system per the manufacturer's protocol. Gene expression was normalized to actin and quantified via the 2^−ΔΔCt^ method. Primer sequences are listed in Table S2.

### 2.13 Chromatin immunoprecipitation quantitative PCR

Chromatin immunoprecipitation (ChIP) assay was conducted using an anti‐ATF3 antibody (ab207434, Abcam) according to the manufacturer's instructions for the ChIP Assay Kit (P2078, Beyotime). Normal rabbit immunoglobulin G (IgG) (AC005, ABclonal) was used as a negative control. The immunoprecipitated DNA was purified using a commercial DNA purification kit (DC301, Vazyme) and subjected to qPCR or PCR analysis. The primer sequences are provided in Table S2.

### 2.14 Determination of GSH and MDA content

The glutathione (GSH) and malondialdehyde (MDA) content in the samples was quantified using commercial assay kits (GSH, S0053; MDA, S0131S; Beyotime) following the manufacturers’ instructions. Briefly, for the GSH assay, samples and reagents were prepared as instructed. The samples were incubated with the total glutathione detection working solution for 5 min, followed by the addition of the NADPH solution. The reaction mixture was then incubated at room temperature for 25 min, and the absorbance was measured at 412 nm using a microplate reader. For MDA determination, sample homogenates were centrifuged to obtain the supernatant. The supernatant was mixed with the freshly prepared MDA detection working solution and heated at 100°C for 15 min. After centrifugation, the absorbance of the supernatant was measured at 532 nm, and the MDA concentration was calculated based on the absorbance values.

### 2.15 Iron content determination

The iron content was measured using the iron assay kit (ab83366, Abcam). In brief, following homogenization and centrifugation of the samples, the supernatant was collected. An iron‐reducing agent and iron buffer were added to the supernatant. After thorough mixing, the mixture was incubated at 37°C for 30 min. Subsequently, 100 µl of an iron probe was added, and the reaction was incubated in the dark at 37°C for 1 h. The absorbance at 593 nm was measured using a microplate reader.

### 2.16 Transmission electron microscope

The mice were transcardially perfused with 2.5% glutaraldehyde. Cortical tissues were dissected along the sagittal plane into 1 mm^3^ cubes and fixed overnight at 4°C in 2.5% glutaraldehyde. The samples were then post‐fixed with 1% osmium tetroxide for 2 h at room temperature, protected from light. Following dehydration and embedding, ultrathin sections (70 nm) were prepared using an ultramicrotome. The sections were stained with 2% uranyl acetate in ethanol for 10 min in the dark, followed by staining with lead citrate for 10 min in a CO_2_‐free environment. Finally, the sections were observed and imaged using a transmission electron microscope (HITACHI). Cultured cells were processed identically.

### 2.17 Adeno‐associated virus mediated ATF3 knockdown

To achieve ATF3 knockdown in vECs, we used the adeno‐associated virus (AAV) ‐BI30 serotype for its specific and efficient transduction of endothelial cells within the central nervous system, including the venous compartment.^30^ The pAAV‐TIE1p‐MCS‐mCherry‐miR30shRNA (ATF3/NC)‐WPRE was obtained from OBiO Technology (Shanghai, China). AAV‐BI30 was intravenously administered to 10‐month‐old APP23 mice at a dose of 3 × 10^1^ ^1^ vg per mouse. The sequences of shRNA were provided in Table S3.

### 2.18 Morris water maze experiment

Spatial learning and memory function of mice was assessed by the Morris water maze test. The test was initiated 3 weeks after AAV injection and consisted of a 5‑day acquisition phase followed by a 1‑day probe trial. The apparatus consisted of a circular pool (120 cm in diameter) filled with water rendered opaque by titanium dioxide and maintained at 22–25°C. A hidden platform (1 cm below the water surface) was placed in the target quadrant. During the acquisition phase, mice underwent four trials per day from different starting quadrants. In each trial, the mouse was allowed 60 s to locate the submerged platform. If it failed to find the platform within the allotted time, it was gently guided to the platform and allowed to remain there for 20 s. The movement trajectories and the latency to find the platform were recorded using the ANY‐maze software (Stoelting, USA). On the day of the probe trial, the platform was removed, and each mouse was allowed to swim freely for 60 s. The following parameters were recorded: swimming trajectory, latency to the first crossing of the former platform location, time spent in the target quadrant and number of platform crossings.

### 2.19 Cell culture and Aβ1‐40 treatment

The bEnd.3 endothelial cell line was provided by Professor Xufang Sun.^31^ Cells were cultured in Dulbecco's Modified Eagle Medium (DMEM; PM150210, Pricella) supplemented with 10% fetal bovine serum (G8003, Servicebio) and 1% penicillin/streptomycin (G4003, Servicebio) at 37°C in a humidified 5% CO_2_ incubator. The medium was changed every 2–3 days, and cells were passaged upon reaching confluence. For experiments, cells at an appropriate confluence were treated with either 0.02% DMSO (vehicle control) or 1 µM Aβ1‐40 oligomers for 12 h. Detailed information on the key reagents is provided in Table S4.

### 2.20 Lentivirus transfection

The lentivirus carrying shRNA targeting ATF3 (sequences targeting mouse ATF3 provided in Table S3) was obtained from Wuhan Paiwei Biotechnology Co., Ltd. For transduction, bEnd.3 cells at an optimal growth stage were incubated with the viral suspension. After 12 h, the viral medium was replaced with fresh complete medium. Cells were then cultured for an additional 36 h prior to subsequent experimental treatments.

### 2.21 Statistical analysis

Statistical analyses were performed using GraphPad Prism (version 8.0.2). Data are presented as mean ± standard deviation (SD). Normality was assessed using the Shapiro–Wilk test. An independent‐samples *t*‐test was used for comparisons between two groups, and one‐way or two‐way analysis of variance (ANOVA), followed by an appropriate multiple‐comparisons test, was employed for comparisons among multiple groups. A two‐sided *p‐*value < 0.05 was considered statistically significant.

## 3 RESULTS

### 3.1 Single‐cell transcriptomic profiling of cerebral microvessels in APP23 mice

**FIGURE 1:**
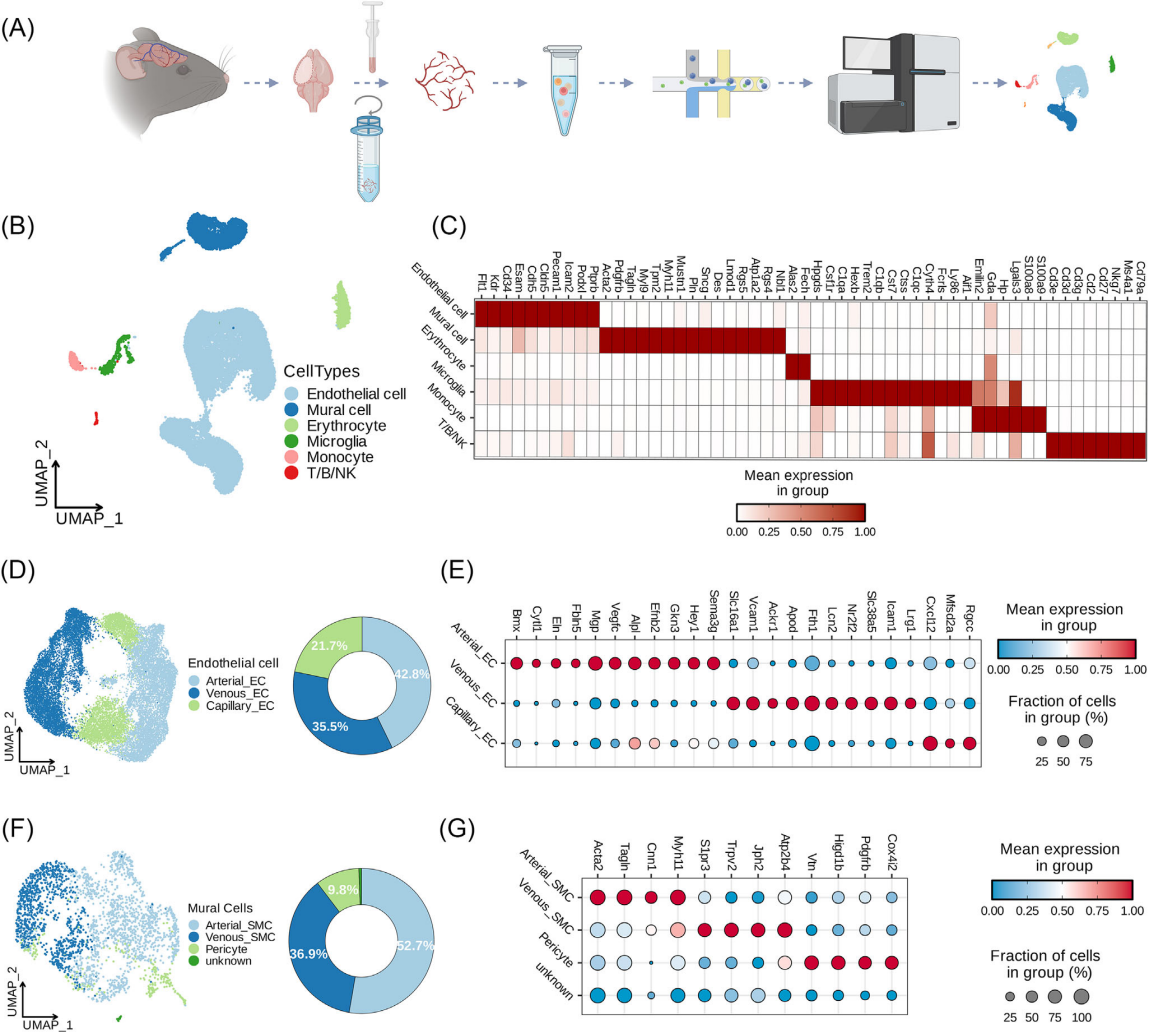
Single‐cell transcriptomic profiling of cerebral microvessels in APP23 mice. (A) Schematic overview of the experimental design. Created with BioRender. (B) Uniform Manifold Approximation and Projection (UMAP) visualization of all cells, colored by major cell types. (C) Heatmap displaying cell type ‐specific marker genes from the single‐cell RNA sequencing (scRNA‐seq) analysis. (D, F) Left panel: UMAP plots of endothelial cells (D) and mural cells (F), colored by subpopulation. Right panel: Pie charts illustrating the proportional distribution of each subpopulation, with colors matching the UMAP plots. (E, G) Dot plots showing expression levels of marker genes for each endothelial (E) and mural (G) subpopulation.

APP23 transgenic mice were used as the murine model of CAA. Genotyping results are shown in Figure S1. CAA onset in this mouse model is reported at 9–12 months of age.^32^ To characterize the transcriptomic features of different cell types in the cerebral microvasculature during early CAA pathogenesis, we performed scRNA‐seq on cortical microvessels from 11‐month‐old male APP23 and WT mice (Figure 1). The schematic diagram for sample preparation and scRNA‐seq is depicted in Figure 1A. After quality control and batch effect correction, a total of 25,919 single cells were obtained and clustered into six major cell types based on well‐established markers,^33^, ^34^, ^35^ including endothelial cells (ECs), mural cells, microglia, monocytes, T/B/NK cells, and erythrocytes (Figure 1B, C and Figure S2). ECs constituted the predominant population in cortical microvessels, followed by mural cells. Subset analysis was then performed on these two major cell types to identify their respective subpopulations. Among ECs, 19,623 cells were classified into three subtypes, with arterial ECs representing 42.8%, followed by venous ECs (35.5%) and capillary ECs (21.7%) (Figure 1D, E). For mural cells, 3330 cells were grouped into three subtypes, of which arterial smooth muscle cell accounted for 52.7% (Figure 1F, G).

### 3.2 Dysregulated iron metabolism in microvascular cells of APP23 mice

**FIGURE 2:**
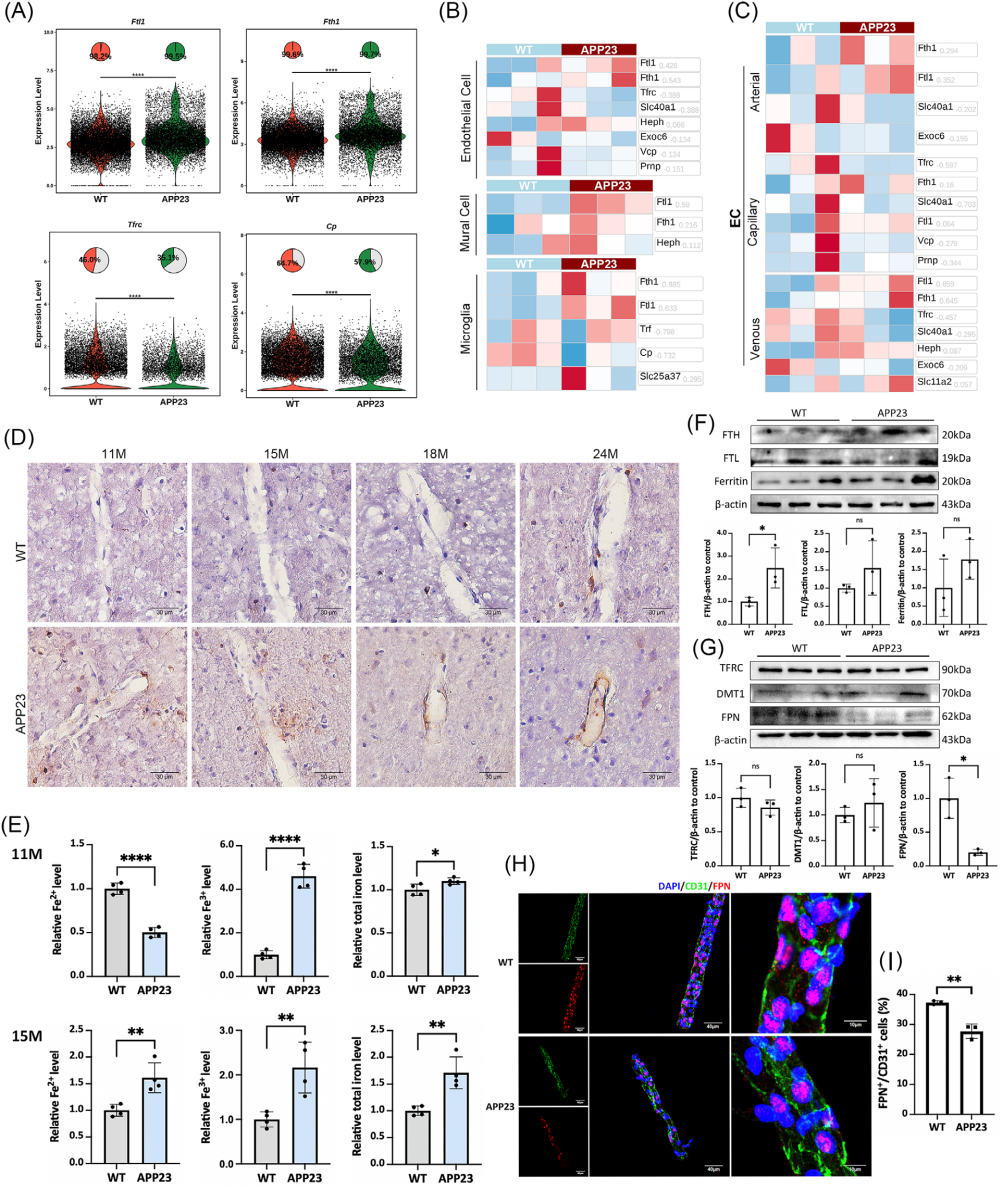
Dysregulated iron metabolism in microvascular cells of APP23 mice. (A) Violin plots displaying expression levels of *Ftl1*, *Fth1*, *Tfrc*, and *Cp* in the cerebral microvascular cells of APP23 versus wild‐type (WT) mice. The percentage of cells expressing each gene is indicated in the overlaid pie charts. (B, C) Heatmaps showing the expression profiles of selected genes involved in iron metabolism across different cell types in APP23 and WT mice, with the logFC labeled numerically in gray after each gene name. (D) Representative images of iron deposition in cortical microvessels of APP23 and WT mice at indicated ages. Iron was visualized by DAB‐enhanced Perls’ Prussian blue staining, which appears as a brown precipitate. Scale bar = 30 µm. (E) Box plots of relative Fe^2^^+^, Fe^3^^+^, and total iron levels in cortical microvessels of 11 (top) and 15‐month‐old (bottom) APP23 versus WT mice. *N* = 4 per group. (F, G) Western blot analysis of the protein levels of ferritin (Ferritin, FTH, FTL) (F) and key iron transporters (TFRC, DMT1, FPN) (G) in microvessels from 11‐month‐old APP23 and WT mice. Upper panels: Representative immunoblot images. Lower panels: Box plots of densitometric quantifications of indicated proteins normalized to β‐actin. (H, I) Representative immunofluorescence images (H) and quantification (I) of co‐staining for CD31 (green) and FPN (red) in cortical endothelial cells. Scale bar = 40 µm. *N* = 3 per group for quantitative analysis. Data are expressed as mean ± SD. *p < 0.05; **p < 0.01; ****p < 0.0001; ns, not significant; unpaired Student's *t*‐test (E, F, G, I).

Given the close link between iron metabolism and ferroptosis and its prominent role in neurological pathology, we next examined iron metabolism gene signatures. According to the DEGs analysis, we found that APP23 mice exhibited upregulation of *Ftl1* and *Fth1*, alongside downregulation of *Tfrc* and *Cp* (Figure 2A). Examination at single‐cell resolution revealed that this iron metabolism dysregulation was present across three major cell types including ECs, mural cells, and microglia. Of note, ECs displayed the greatest number of dysregulated features (Figure 2B). Among endothelial subpopulations, the venous subset was the most affected, with the highest number of dysregulated iron metabolism genes in APP23 mice, including elevated expression of *Fth1* but reduced expression of *SLC40A1* compared to the WT group (Figure 2C).

Enhanced Prussian blue staining confirmed increased iron deposition in the cortical microvessels of APP23 mice. This deposition was observed from the early disease stage (11 months) through advanced age (24 months) (Figure 2D). Corroborating this observation, quantitative analysis demonstrated elevated Fe^3^^+^ and total iron in microvessels at 11 months, which progressed to include significantly increased Fe^2^^+^ levels by 15 months (Figure 2E). Notably, this iron dysregulation was primarily localized to the vasculature, as the cortical tissue showed no pronounced increase in Fe^2^^+^ despite only slight elevations in Fe^3^^+^ and total iron (Figure S3). Subsequent Western blot analysis of microvessels from 11‐month‐old mice for ferritin (Ferritin, FTH, FTL) (Figure 2F) and major iron transporters (TFRC, DMT1, FPN) (Figure 2G) revealed increased FTH and decreased FPN expression in APP23 mice, corroborating the scRNA‐seq findings. Immunofluorescence staining verified the reduced FPN expression in cortical ECs (Figure 2H,I). These results demonstrate dysregulated iron metabolism in the microvascular cells of APP23 mice.

### 3.3 scRNA‐seq identifies a preferential vulnerability to ferroptosis in venous ECs

**FIGURE 3:**
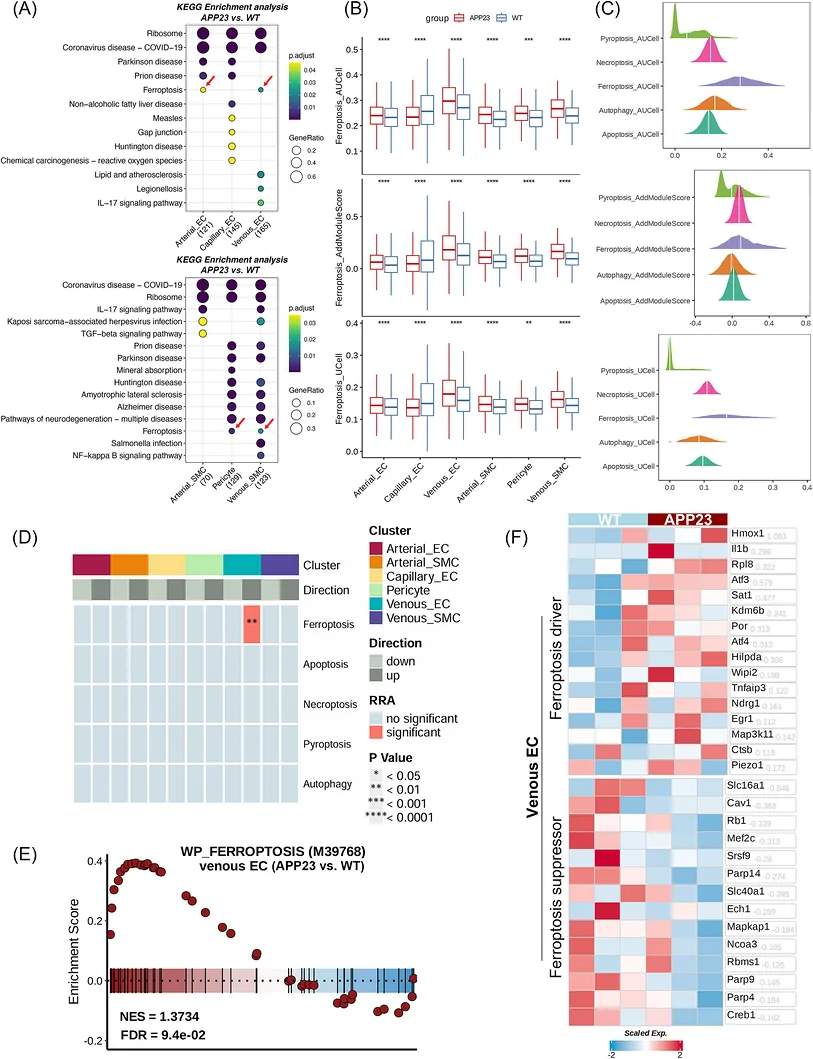
Single‐cell RNA sequencing (scRNA‐seq) identifies a preferential vulnerability to ferroptosis in venous endothelial cells. (A) Dot plots of Kyoto Encyclopedia of Genes and Genomes (KEGG) pathway enrichment analysis showing ferroptosis enrichment (highlighted by red arrows) in the indicated subpopulations of endothelial cells (upper panel) and mural cells (lower panel). (B) Box plots of ferroptosis scores calculated by AUCell (top), AddModuleScore (middle), and UCell (bottom) methods for each subpopulation in APP23 and wild‐type (WT) mice. (C) Ridgeline plots showing the distribution of five programmed cell death (PCD) scores in venous endothelial cell cluster. (D) Heatmap displaying the enrichment of PCD pathways across different cerebrovascular cell clusters. (E) Gene set enrichment analysis (GSEA) indicating upregulation of ferroptosis in venous endothelial cell cluster of APP23 mice. (F) Heatmaps showing the expression profiles of ferroptosis‐related genes in APP23 and WT mice, with the logFC labeled numerically in gray after each gene name.

Next, we performed KEGG enrichment analysis to identify the biological pathways altered in APP23 mice. The results indicated that the ferroptosis signaling pathway was enriched in arterial ECs, venous ECs, pericytes, and venous smooth muscle cells (Figure 3A). To robustly assess ferroptosis activity across these subpopulations, three scoring algorithms were applied. All three methods consistently showed that venous ECs exhibited the most pronounced increase in ferroptosis activity of APP23 mice (Figure 3B). Moreover, among the five well‐established PCD pathways, ferroptosis demonstrated the highest activity in venous EC cluster (Figure 3C).

To further validate the robustness of these findings, we employed the rank‐based single‐cell enrichment method irGSEA, which integrates six gene set scoring techniques. This analysis confirmed that, among multiple PCD pathways evaluated across different cell subtypes, ferroptosis was the only one significantly and specifically enriched in venous ECs (Figure 3D). Consistently, GSEA verified the upregulation of the ferroptosis pathway in venous ECs of APP23 mice (Figure 3E). Furthermore, using gene sets from the FerrDb V2 database, we observed distinct expression patterns in the venous ECs of APP23 mice: several ferroptosis drivers (e.g., *HMOX1*, *ATF3*, *SAT1*, *RPL8*) were upregulated, while key ferroptosis suppressors (e.g., *CAV1*, *RB1*, *SLC40A1, NCOA3*) were downregulated (Figure 3F).

### 3.4 In vivo analyses support early and progressive ferroptosis‐related alterations in cortical venular ECs during CAA development

**FIGURE 4:**
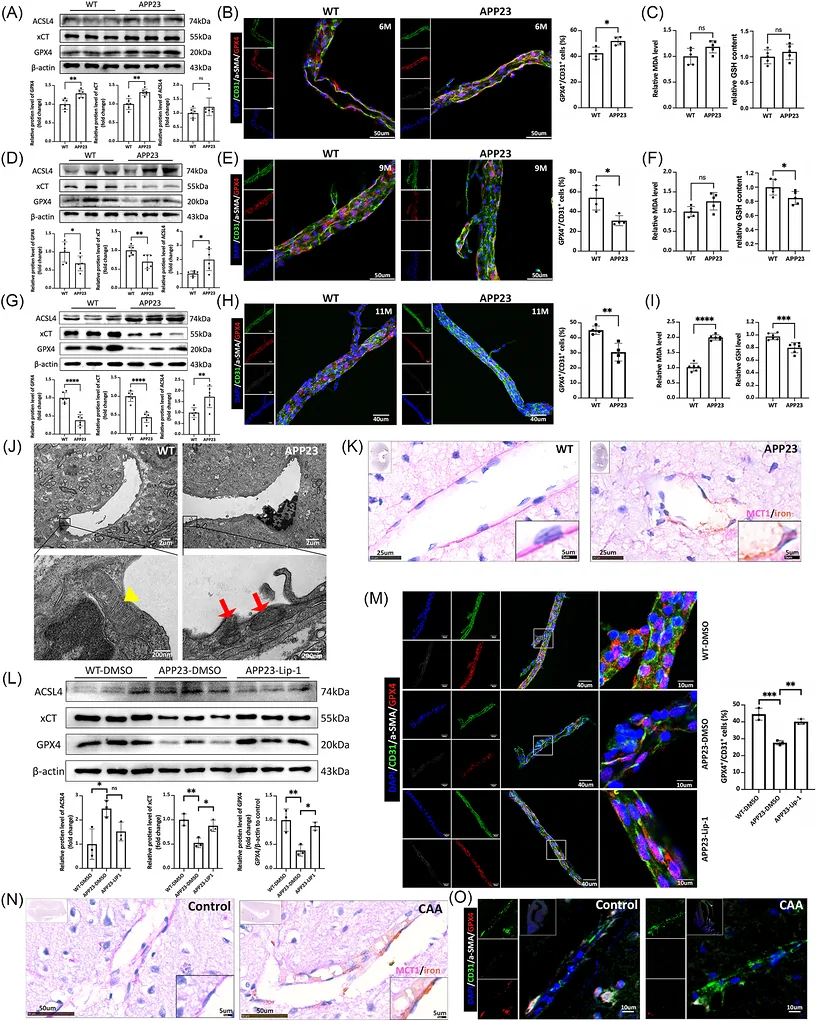
Ferroptosis in cortical venular endothelial cells (vECs) during the early stage of cerebral amyloid angiopathy (CAA). (A, D, G) Western blot analysis of ACSL4, xCT, and GPX4 protein levels in cortical microvessels from APP23 and wild‐type (WT) mice at 6 months (A), 9 months (D), and 11 months (G). For each age, representative immunoblot images are shown in the upper panels, and the corresponding protein levels normalized to β‐actin are shown in the lower panels. *N* = 6 per group for quantitative analysis. (B, E, H) Immunofluorescence analysis of GPX4 expression in cortical vECs from APP23 and WT mice at 6 months (B), 9 months (E), and 11 months (H). For each age, representative immunofluorescence images are shown in the left panels, and quantification of the proportion of GPX4‐positive vECs is shown in the right panels. Scale bars, 50 µm in (B) and (E) and 40 µm in (H). *N* = 4 per group for quantitative analysis. (C, F, I) Box plots showing relative GSH and MDA levels in cortical microvessels from APP23 mice, normalized to the corresponding age‐matched WT controls, at 6 months (C), 9 months (F), and 11 months (I). *N* = 5 per group in (C) and (F), and *N* = 6 per group in (I). (J) Transmission electron microscopy showing ferroptosis‐associated mitochondrial alterations in cortical vECs from 11‐month‐old APP23 mice. The yellow arrowhead indicates a mitochondrion with normal morphology, and the red arrow point to mitochondria displaying features characteristic of ferroptosis. Scale bar = 200 nm. (K) Representative images of MCT1 immunohistochemistry combined with DAB‐enhanced Perls’ Prussian blue staining in brain sections from 11‐month‐old APP23 and WT mice. MCT1‐positive vECs are stained red with AEC, whereas iron deposits are stained brown. Scale bar = 25 µm. (L) Western blot analysis of ACSL4, xCT, and GPX4 protein levels in cortical microvessels from the indicated mouse groups. Representative immunoblot images are shown in the upper panel, and box plots showing the corresponding densitometric quantification normalized to β‐actin are shown in the lower panel. (M) Immunofluorescence analysis of GPX4 expression in cortical vECs from the indicated mouse groups. Representative immunofluorescence images are shown in the left panel, and the quantification of the proportion of GPX4‐positive vECs is shown in the right panel. Scale bar = 40 µm. *N* = 3 per group for quantitative analysis. (N) Representative images of MCT1 immunohistochemistry combined with DAB‐enhanced Perls’ Prussian blue staining in cortical tissues from a patient with CAA and a control subject. MCT1‐positive vECs are stained red with AEC, whereas iron deposits are stained brown. Scale bar = 50 µm. (O) Representative images of triple immunofluorescence staining for CD31 (green), α‐SMA (white), and GPX4 (red) in cortical venules from a patient with CAA and a control subject. Scale bar = 10 µm. Data are expressed as mean ± SD. *p < 0.05; **p < 0.01; ***p < 0.001; ****p < 0.0001; ns, not significant; unpaired Student's *t*‐test (A–I); one‐way ANOVA followed by Dunnett's multiple comparisons test (L, M).

Given that the venous EC cluster was identified by scRNA‐seq of cortical microvessels, we examined cortical venular endothelial cells (vECs) in 6‐ to 11‐month‐old APP23 mice to validate the scRNA‐seq findings and characterize ferroptosis‐related alterations during early CAA development (Figure 4). At 6 months, we observed increased expression of xCT and GPX4 in microvessels, together with mild elevations in ACSL4 and MDA levels that did not reach statistical significance. Consistently, immunofluorescence staining demonstrated increased GPX4 expression in vECs (Figure 4A–C). These findings may indicate a compensatory activation of anti‐ferroptotic defenses in response to potential stressors such as soluble amyloid‐β, with trends toward increased ACSL4 and MDA levels suggesting a subtle rise in lipid peroxidative pressure. At 9 months, ferroptosis‐related alterations in microvessels became more evident, characterized by decreased xCT and GPX4 expression, increased ACSL4 expression, and decreased GSH levels. Immunofluorescence staining revealed that GPX4 expression was reduced in vECs at this stage (Figure 4D–F). At 11 months, the ferroptotic phenotype was further intensified, accompanied by impaired antioxidant defense and increased lipid peroxidation. Quantitative analysis of cortical microvessels additionally demonstrated a significant elevation of MDA levels in APP23 mice compared with age‐matched WT controls. The proportion of GPX4‐positive endothelial cells (GPX4^+^/CD31^+^) within cortical venules of APP23 mice was further reduced (Figure 4G–I). To determine whether these molecular changes were accompanied by characteristic ultrastructural and histopathological features, we examined 11‐month‐old APP23 mice and age‐matched WT controls. Compared with WT controls, vECs from APP23 mice exhibited smaller, electron‐dense mitochondria with reduced cristae on transmission electron microscopy (TEM) (Figure 4J). MCT‐1 (a known marker of vECs^36^) immunohistochemistry combined with enhanced Perls' Prussian blue staining revealed more prominent iron deposition in cortical venules of APP23 mice (Figure 4K). Importantly, treatment with the ferroptosis inhibitor Lip‐1 in 10‐month‐old APP23 mice restored xCT and GPX4 expression in cortical microvessels and increased the proportion of GPX4^+^/CD31^+^ endothelial cells in cortical venules (Figure 4L,M). Preliminary examination of cortical tissue from patients with CAA also revealed venular iron deposition and a possible reduction in GPX4 expression in vECs, although no quantitative analysis was performed (Figure 4N,O and Figure S4).

Collectively, these findings support the susceptibility of vECs to ferroptosis in APP23 mice and suggest that ferroptosis is an early and progressive process during CAA development.

### 3.5 Aβ1‐40 triggers ferroptosis in vECs

**FIGURE 5:**
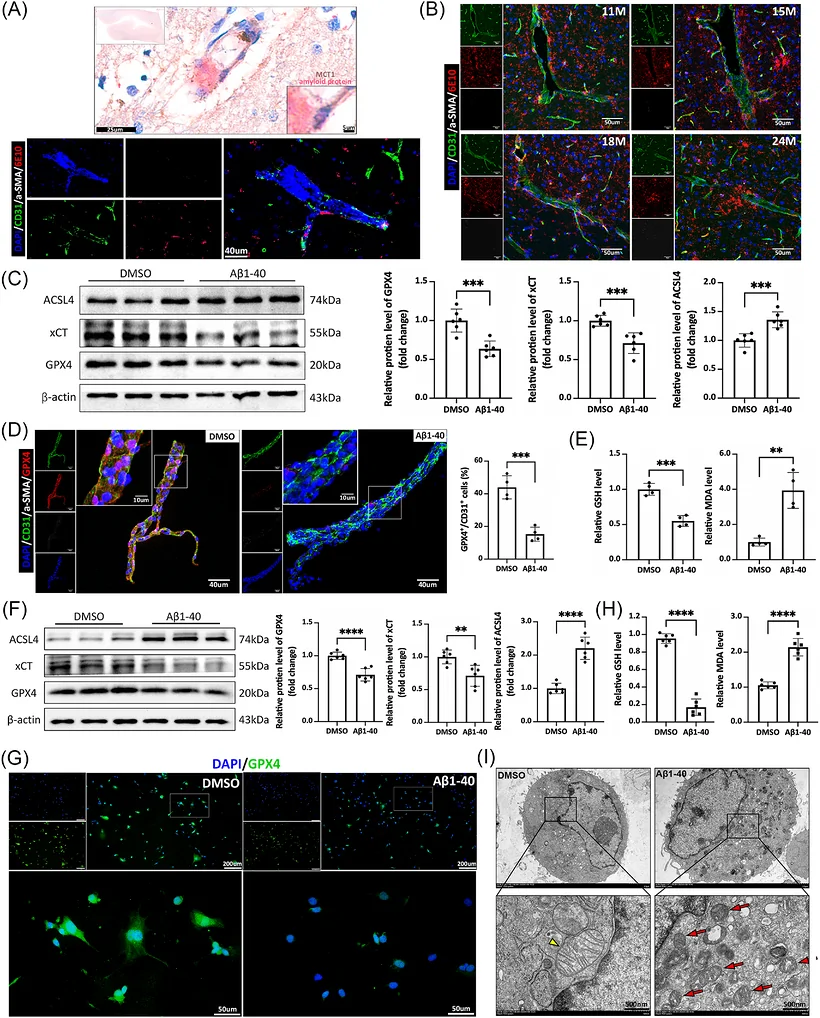
Aβ1‐40 induces ferroptosis in venular endothelial cells (vECs). (A, B) Cortical venular amyloid‐beta (Aβ) deposition in CAA. (A) Upper panel: Immunohistochemistry for MCT1 (brown) combined with Congo red (red) staining on an occipital venule from a cerebral amyloid angiopathy (CAA) patient. Scale bar = 25 µm.  Lower panel: Representative immunofluorescence images of CD31 (green), alpha‐smooth muscle actin (α‐SMA) (white), and 6E10 (red) co‐staining in an occipital venule from a CAA patient. Scale bar = 40 µm. (B) Representative immunofluorescence images of CD31 (green), α‐SMA (white), and 6E10 (red) co‐staining in cortical venules of brain sections from APP23 mice across 11 to 24‐month age. Scale bar = 50 µm. (C–E) Ferroptosis‐related changes in vECs induced by Aβ1‐40. (C) Representative immunoblot images (left panel) and analysis (right panel) of ACSL4, xCT, and GPX4 in microvessels stimulated with DMSO or Aβ1‐40. *N* = 6 per group for analysis. (D) Representative immunofluorescence images (left panel) and analysis (right panel) of CD31 (green), α‐SMA (white), and GPX4 (red) co‐staining in venules following DMSO or Aβ1‐40 treatment. Scale bar = 40 µm. *N* = 4 per group for analysis. (E) The relative levels of GSH and MDA in microvessels treated with DMSO or Aβ1‐40. *N* = 4 per group. (F–I) Ferroptosis‐related changes in bEnd.3 cells induced by Aβ1‐40. (F) Representative immunoblot images (left panel) and analysis (right panel) of ACSL4, xCT, and GPX4 in bEnd.3 cells stimulated with DMSO or Aβ1‐40. *N* = 6 per group for analysis. (G) Representative immunofluorescence images of GPX4 (green) in bEnd.3 cells treated with DMSO or Aβ1‐40. Scale bar = 50 µm. (H) The relative levels of GSH and MDA (*N* = 6 per group) in bEnd.3 cells treated with DMSO or Aβ1‐40. (I) Transmission electron microscopy showing the morphology of mitochondria in bEnd.3 cells treated with DMSO or Aβ1‐40. The yellow arrowhead indicates a mitochondrion with normal morphology, and the red arrows point to mitochondria displaying features characteristic of ferroptosis. Scale bar = 500 nm. Data are expressed as mean ± SD. **p < 0.01; ***p < 0.001; ****p < 0.0001, unpaired Student's *t*‐test (C–F, H).

Considering the important role of Aβ in inducing ferroptosis in AD,^37^ we hypothesized that ferroptosis in the vECs of CAA might likewise be associated with Aβ accumulation. To test this hypothesis, we performed two independent staining approaches on postmortem brain sections from sporadic CAA patients: an MCT1/Congo red co‐staining and a triple immunofluorescence of CD31, a‐SMA, and 6E10. Both approaches confirmed Aβ deposition in the cortical venules (Figure 5A, Figure S4). In addition, immunofluorescence staining of brain sections from APP23 mice across 11 to 24 months of age revealed the presence of Aβ deposits in cortical venules at all time points examined (Figure 5B). In order to investigate the impact of Aβ on vECs, we treated both aseptically isolated cortical microvessels from WT mice and cultured bEnd.3 cells with 1 µM oligomeric Aβ1‐40 for 6 h and 12 h, respectively. The Aβ1‐40 concentration and treatment duration were determined based on previous literature^19^, ^38^, ^39^ and preliminary experiments (Figure S5). In microvessels, Western blot analysis showed decreased protein levels of the ferroptosis suppressors GPX4 and xCT, alongside increased levels of the ferroptosis promoter ACSL4 in Aβ1‐40 stimulation group (Figure 5C). Immunofluorescence staining confirmed a marked decrease in the proportion of GPX4 positive vECs following Aβ1‐40 treatment (Figure 5D). Furthermore, biochemical assays demonstrated decreased GSH and increased MDA in Aβ1‐40 treated microvessels (Figure 5E). In bEnd.3 cells, Aβ1‐40 treatment also downregulated the protein levels of GPX4 and xCT while upregulating ACSL4 (Figure 5F). The reduction in GPX4 protein was supported by a corresponding decrease in its immunofluorescence staining intensity (Figure 5G). Simultaneously, Aβ1‐40 stimulation resulted in increased MDA and decreased GSH levels (Figure 5H), suggesting enhanced lipid peroxidation and antioxidant depletion. TEM images showed characteristic ferroptotic changes in mitochondria of Aβ1‐40–treated cells, including mitochondrial shrinkage, increased electron density, and reduction of cristae, compared to DMSO‐treated controls (Figure 5I). These results suggest that Aβ1‐40 stimulation could induce ferroptosis in cerebral vECs.

### 3.6 Aβ1‐40 induces vEC ferroptosis via the ATF3/xCT axis

**FIGURE 6:**
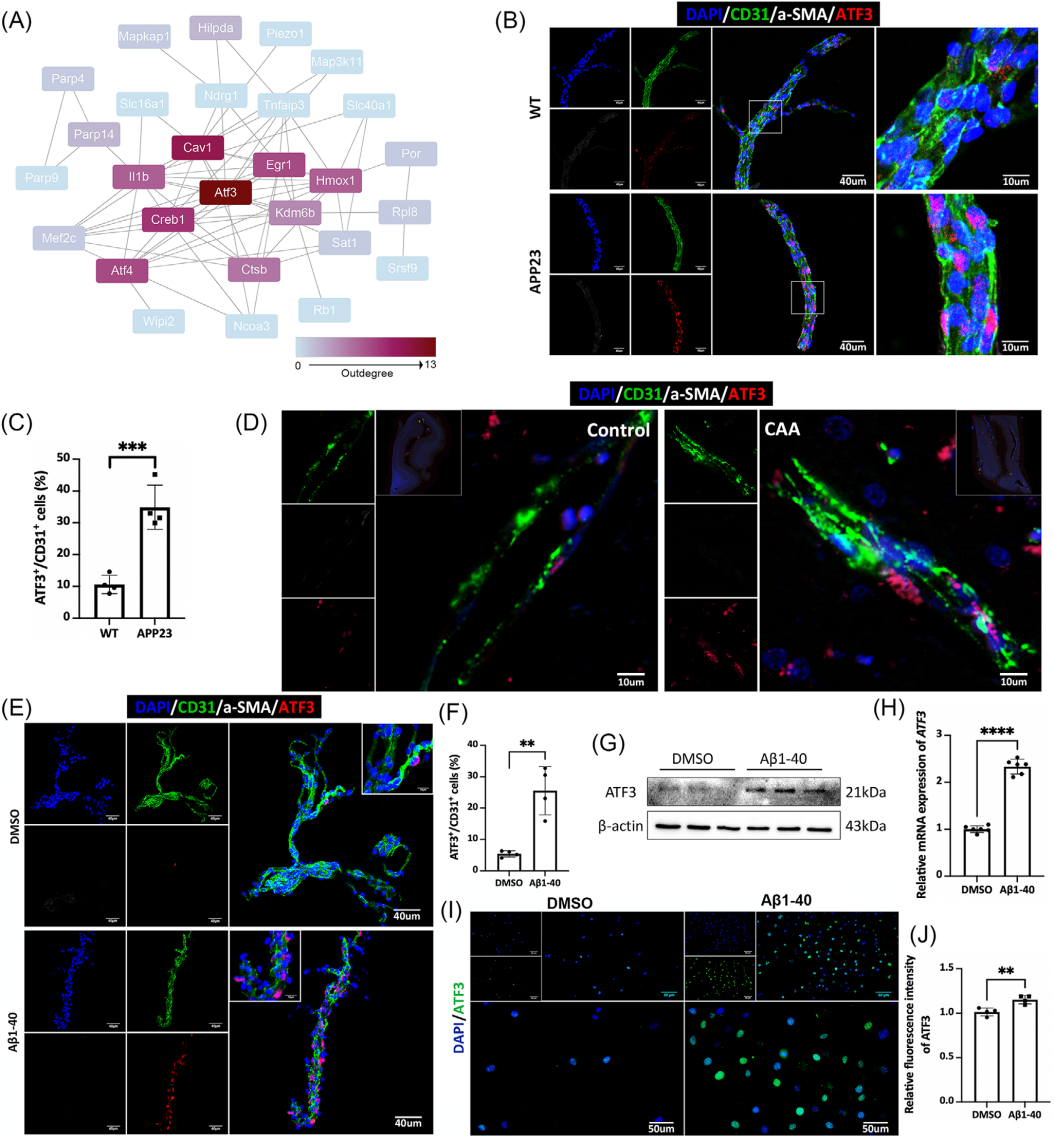
Identification of activating transcription factor 3 (ATF3) as a central hub in the ferroptosis network of venular endothelial cells (vECs) in cerebral amyloid angiopathy (CAA). (A) Protein–protein interaction (PPI) network identifying ATF3 as hub gene. (B) Representative immunofluorescence images of CD31 (green), α‐SMA (white), and ATF3 (red) co‐staining in cortical venules from wild‐type (WT) and APP23 mice, Scale bar = 40 µm. (C) Box plots showing the proportion of ATF3‐positive endothelial cells in cortical venules of WT and APP23 mice. *N* = 4 per group. (D) Representative immunofluorescence images of CD31 (green), α‐SMA (white), and ATF3 (red) co‐staining in cortical venules from CAA patients and controls. Scale bar = 10 µm. (E) Representative immunofluorescence images of CD31 (green), α‐SMA (white), and ATF3 (red) co‐staining in cortical venules from WT mice treated with DMSO or Aβ1‐40. Scale bar = 40 µm. (F) Box plots showing the proportion of ATF3‐positive endothelial cells in cortical venules from WT mice treated with DMSO or Aβ1‐40. *N* = 4 per group. (G) Representative immunoblot images of ATF3 in bEnd.3 cells treated with DMSO or Aβ1‐40. (H) Relative mRNA expression levels of ATF3 in bEnd.3 cells treated with DMSO or Aβ1‐40. *N* = 6 per group. (**I**) Representative immunofluorescence images of ATF3 (green) in bEnd.3 cells treated with DMSO or Aβ1‐40. Scale bar = 50 µm. (J) Box plots showing the fluorescence intensity of ATF3 in bEnd.3 cells treated with Aβ1‐40 relative to the DMSO‐treated control. *N* = 4 per group. Data are expressed as mean ± SD. **p < 0.01; ***p < 0.001; ****p < 0.0001; unpaired Student's *t*‐test (C, F, H, J).

To identify key regulators of Aβ1‐40–induced ferroptosis in CAA vECs, PPI network analysis was performed on differentially expressed ferroptosis drivers and suppressors (sourced from FerrDb V2) derived from the scRNA‐seq dataset of APP23 and WT mice. This analysis pinpointed Activating Transcription Factor 3 (ATF3) as the central hub gene (Figure 6A). This finding was confirmed by multiplex immunofluorescence analysis, which revealed elevated ATF3 expression in the cortical vECs of APP23 mice (Figure 6B,C). In addition, representative staining of CAA patient brain sections showed a similar increase in ATF3 immunoreactivity in cortical vECs (Figure 6D and Figure S4). Furthermore, Aβ1‐40 stimulation significantly upregulated ATF3 expression in both vECs isolated from WT mice (Figure 6E,F) and in cultured bEnd.3 cells (Figure 6G–J), suggesting that Aβ1‐40 directly induces ATF3 expression in vECs.

**FIGURE 7:**
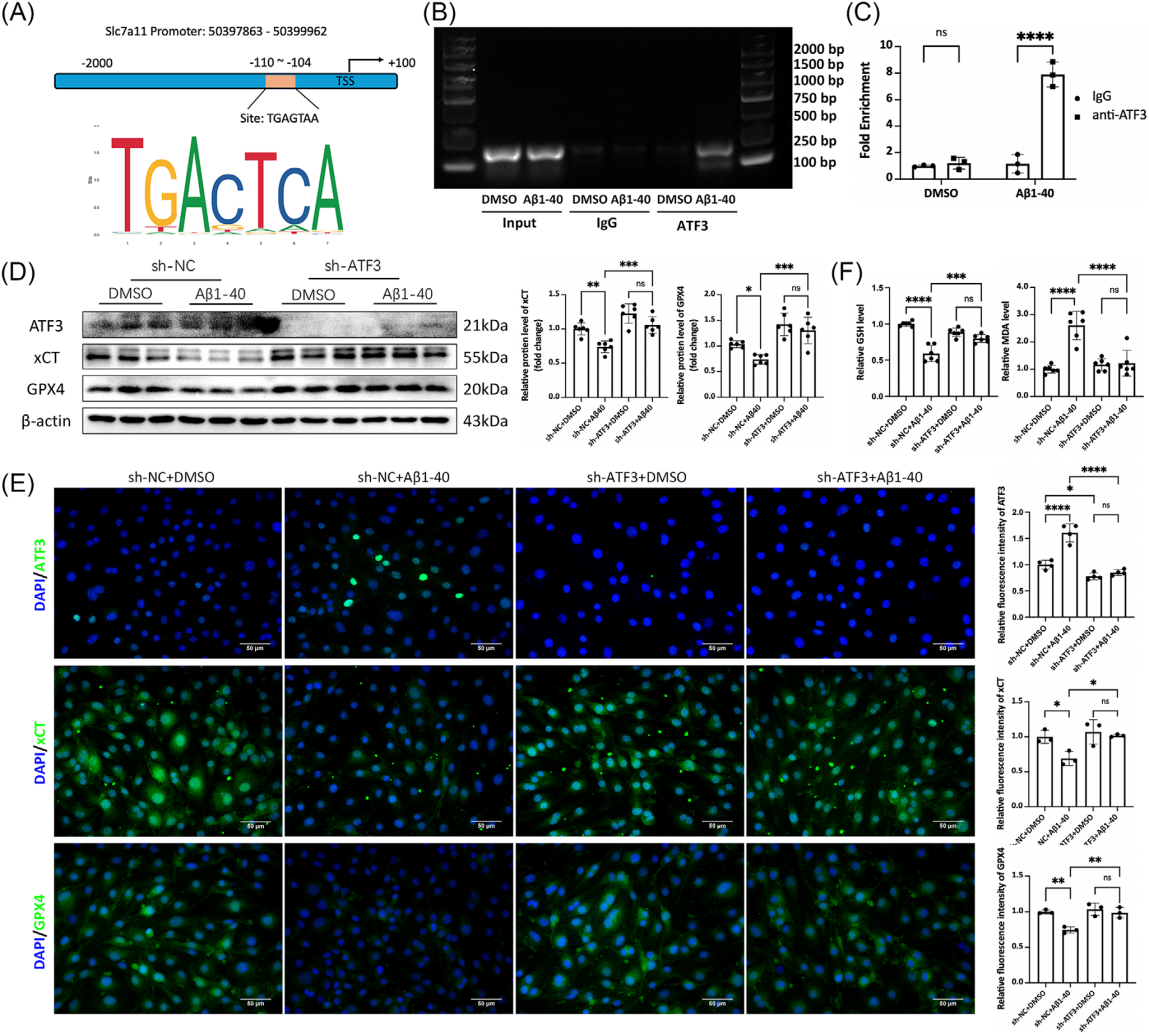
Knockdown of activating transcription factor 3 (ATF3) in bEnd.3 cells attenuates Aβ1‐40–induced ferroptosis by restoring the *Slc7a11*/xCT axis. (A–C) Aβ1‐40 enhances ATF3 enrichment at the *Slc7a11* promoter in bEnd.3 cells. (A) Schematic representation of the mouse *Slc7a11* promoter region and the predicted ATF3‐binding site (upper panel). Motif sequence logo plot of ATF3 from the JASPAR database (lower panel). (B) Representative agarose gel image of chromatin immunoprecipitation quantitative polymerase chain reaction (ChIP‐PCR) products obtained from bEnd.3 cells treated with DMSO or 1 µM oligomeric Aβ1‐40 for 12 h. (C) ChIP‐qPCR analysis showing increased ATF3 enrichment at the predicted *Slc7a11* promoter region following Aβ1‐40 treatment. *N*  =  3 per group. (D) Representative immunoblot images (left panel) and statistical analysis (right panel) of ATF3, xCT and GPX4 in groups with lentivirus treatment. *N* = 6 per group for quantitative analysis. (E) Representative immunofluorescence images (left panel) and quantification of their fluorescence intensity (right panel) for ATF3, xCT, and GPX4 in lentivirus‐treated groups. All three proteins are shown in green. Scale bar = 50 µm. For quantitative analysis, *N* = 4 per group for ATF3 and *N* = 3 per group for xCT and GPX4. (F) Relative levels of GSH and MDA in the indicated groups. *N* = 6 per group. Data are expressed as mean ± SD. *p < 0.05; **p < 0.01; ***p < 0.001; ****p < 0.0001; ns, not significant; two‐way analysis of variance (ANOVA) followed by Sidak's multiple‐comparisons test (C); one‐way ANOVA followed by Tukey's multiple comparisons test (D–F).

A previous study demonstrated that ATF3 induces ferroptosis by transcriptionally repressing *SLC7A11* independently of p53, thereby inhibiting system Xc^−^‐mediated cystine transport and GSH synthesis.^40^ To determine whether this regulatory relationship also occurs in endothelial cells, we performed ChIP‐qPCR analysis to determine ATF3 occupancy at the *Slc7a11* promoter region. In Aβ1‐40–treated bEnd.3 cells, the *Slc7a11* promoter region was significantly enriched in ATF3 immunoprecipitates compared with IgG controls. By contrast, no significant enrichment above the IgG background was observed in untreated cells (Figure 7A–C). These findings indicate that Aβ1‐40 stimulation increases ATF3 binding to the *Slc7a11* promoter in bEnd.3 cells, supporting a role for ATF3 in the transcriptional regulation of *Slc7a11*. We next conducted in vitro and in vivo experiments to investigate whether ATF3 knockdown mitigates Aβ‐associated ferroptotic changes in vECs and restores xCT‐dependent anti‐ferroptotic defenses. For the in vitro study, we used lentivirus‐mediated knockdown to reduce ATF3 expression in bEnd.3 cells. The experiment included four groups: shNC‐DMSO, shNC‐Aβ1‐40, shATF3‐DMSO, and shATF3‐Aβ1‐40. The efficiency of ATF3 knockdown was confirmed by Western blot and qPCR analysis (Figure S6). Western blot analysis demonstrated that in control cells (shNC), Aβ1‐40 treatment significantly upregulated ATF3 expression and downregulated both xCT and GPX4, compared to the DMSO‐treated control group (shNC‐Aβ1‐40 vs. shNC‐DMSO). The downregulation of xCT and GPX4 induced by Aβ1‐40 was abolished in ATF3‐knockdown cells (shATF3‐Aβ1‐40 vs. shATF3‐DMSO) (Figure 7D). Immunofluorescence staining validated the Western blot results, providing visual evidence of ATF3, xCT, and GPX4 expression patterns across the four experimental groups (Figure 7E). Biochemical assays showed that Aβ1‐40 stimulation reduced GSH levels and increased MDA content in shNC cells, and these changes were counteracted by ATF3 knockdown (Figure 7F).

**FIGURE 8:**
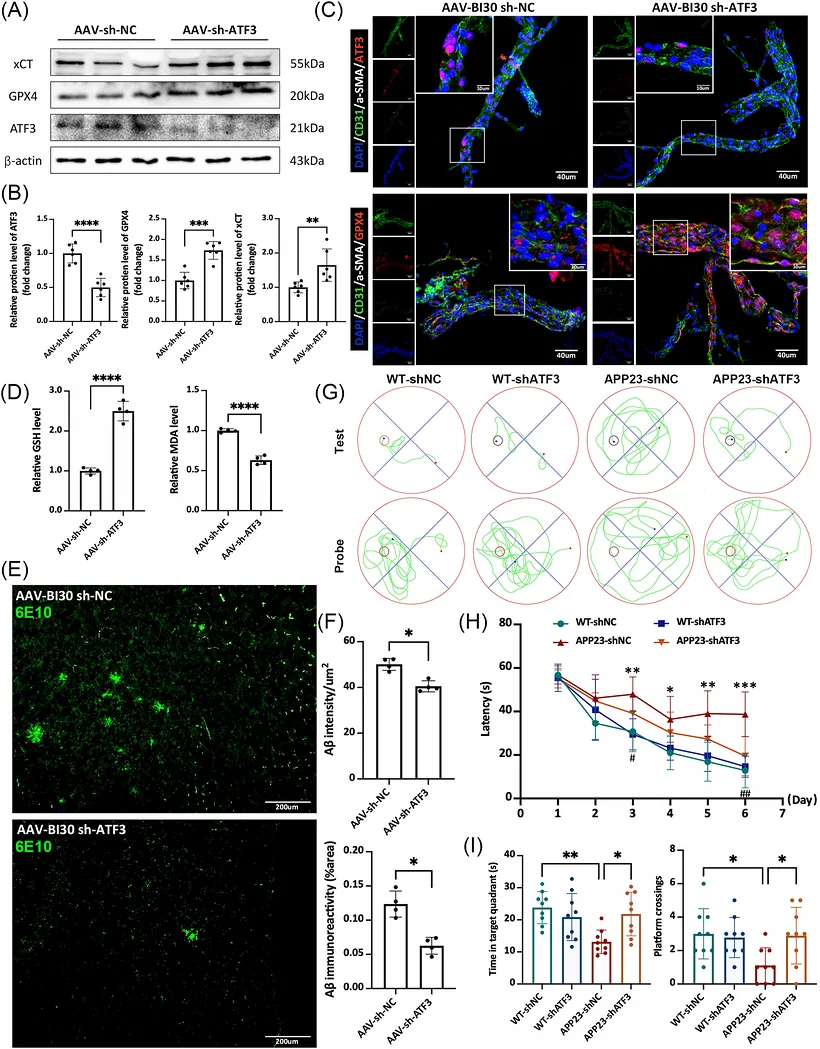
Endothelial activating transcription factor 3 (ATF3) knockdown attenuates ferroptosis‐related alterations in venular endothelial cells (vECs), reduces cerebral amyloid‐beta (Aβ) burden, and ameliorates cognitive deficits in APP23 mice. (A–B). Representative immunoblot images (A) and statistical analysis (B) of ATF3, xCT, and GPX4 in cortical microvessels from adeno‐associated viral vector (AAV) ‐treated APP23 mice. *N* = 6 per group in (B). (C) Representative immunofluorescence images of cortical venules from AAV‐treated APP23 mice. Venules were co‐stained for either ATF3 (red, top) or GPX4 (red, bottom), along with alpha‐smooth muscle actin (α‐SMA) (white) and CD31 (green). Scale bar = 40 µm. (D) Relative levels of GSH and MDA in cortical microvessels from AAV‐treated APP23 mice. *N* = 4 per group. (E, F) Knockdown of ATF3 in cerebral endothelial cells alleviates Aβ pathology in APP23 mice. (E) Representative immunofluorescence image showing Aβ deposition (stained with 6E10, green) in the cortex of AAV‐treated APP23 mice. Scale bar = 200 µm. (F) Quantification of cortical Aβ load, measured as both the mean Aβ fluorescence intensity per µm^2^ (upper panel) and the percentage of Aβ‐immunoreactive area (% Area) (lower panel). *N* = 4 per group. (G) Representative trajectory diagrams of water maze acquisition and probe tests in WT‐shNC, WT‐shATF3, APP23‐shNC and APP23‐shATF3 groups. (H) Analysis of escape latency in the Morris water maze acquisition phase among WT‐shNC, WT‐shATF3, APP23‐shNC, and APP23‐shATF3 groups. *N* = 9 per group. * Indicates WT‐shNC versus APP23‐shNC; # indicates APP23‐shNC versus APP23‐shATF3. *p < 0.05, **p < 0.01, ***p < 0.001, #*p* < 0.05, ##*p* < 0.01. (I) Statistical analysis of the Morris water maze probe test. Time spent in the target quadrant (left). Number of platform crossings (right). Data are from WT‐shNC, WT‐shATF3, APP23‐shNC, and APP23‐shATF3 groups. *N* = 9 per group. Data are expressed as mean ± SD. *p < 0.05; **p < 0.01; ***p < 0.001; ****p < 0.0001; ns, not significant; unpaired Student's *t*‐test (B, D, F); two‐way analysis of variance (ANOVA) followed by Tukey's multiple comparisons test (H); one‐way ANOVA followed by Tukey's multiple comparisons test (I). shNC, *ATF3* negative AAV; shATF3, AAV‐sh*‐ATF3*.

For in vivo experiment, 10‐month‐old APP23 mice received an intravenous injection of AAV‐BI30, which was engineered to selectively reduce ATF3 expression in cerebral vascular ECs including the venular compartment. Four weeks after injection, AAV transduction in cerebral vascular ECs was confirmed by colocalization of red fluorescence with the endothelial marker CD31 (Figure S7). Western blot analysis of isolated cortical microvessels showed that AAV‐mediated ATF3 knockdown significantly reduced ATF3 protein levels and upregulated xCT and GPX4 expression in APP23 mice, compared to control mice (Figure 8A,B). Immunofluorescence staining further confirmed the downregulation of ATF3 and concomitant upregulation of GPX4 in vECs at the cellular level (Figure 8C). In addition, ATF3 knockdown significantly decreased MDA content and increased GSH levels in microvessels (Figure 8D).

Collectively, these results demonstrate that knocking down ATF3 expression in vECs alleviates ferroptotic changes and restores xCT‐dependent anti‐ferroptotic defenses.

### 3.7 Endothelial ATF3 knockdown ameliorates Aβ pathology and cognitive deficits in APP23 mice

Studies indicate that functionally competent venules contribute to Aβ clearance.^41^, ^42^ The attenuation of ferroptotic changes in vECs following endothelial ATF3 knockdown therefore prompted us to examine whether this intervention also influenced cerebral Aβ burden in APP23 mice. Immunofluorescence analysis revealed a significant reduction in Aβ fluorescence intensity per unit area and Aβ immunoactivity (% area) in APP23 mice with ATF3 knockdown compared to the control group (Figure 8E,F), indicating a reduced cerebral Aβ burden.

Cognitive function was subsequently assessed using the Morris water maze. Compared with WT mice, control AAV‐treated APP23 mice showed impaired spatial learning and memory, with longer escape latencies, less time spent in the target quadrant, and fewer platform crossings during the probe trial. Endothelial ATF3 knockdown significantly improved these metrics: It shortened escape latency, increased target quadrant time, and raised the number of platform crossings (Figure 8G–I).

Together, these results indicate that ATF3 knockdown in cerebrovascular endothelial cells, including the venular compartment, reduces cerebral Aβ burden and ameliorates cognitive deficits in APP23 mice.

## 4 DISCUSSION

This study identified a previously overlooked vascular component in the pathophysiology of CAA, emphasizing the role of venular endothelial injury in disease development. Specifically, we found that vECs underwent ferroptosis during the early stage of CAA. This process was associated with Aβ1‐40–induced activation of the ATF3/xCT axis. Knockdown of ATF3 in cerebrovascular endothelial cells alleviated ferroptosis‐related alterations in vECs, reduced cerebral Aβ load, and ameliorated cognitive deficits in APP23 mice. These results suggest an interplay between Aβ accumulation and vEC ferroptosis and highlight venular endothelial ferroptosis as a promising therapeutic target for CAA.

Endothelial PCD is an important factor in vascular dysfunction.^43^ In our study, single‐cell transcriptomic profiling of cortical microvessels from 11‐month‐old APP23 mice revealed that ferroptosis signatures were most prominently enriched among the examined PCD pathways in venous endothelial cluster. Further validation across different ages spanning the early stage of CAA, including 6, 9, and 11 months, demonstrated that the expression of ferroptosis‐associated molecules was already dysregulated at 6 months. At 9 months, ferroptosis‐related protein alterations became evident, whereas more pronounced ferroptotic changes of vECs were observed at 11 months. These findings indicate that ferroptosis may be an early and progressive process underlying vEC dysfunction during CAA development.

Venular Aβ deposition is an important but underappreciated pathological feature of CAA. Indeed, Michaud et al. reported that small Aβ aggregates on veins seem to precede their deposition in arteries,^5^ suggesting that venous Aβ accumulation may serve as an early vascular stressor. In our study, Aβ1‐40 stimulation induced ferroptosis‐related alterations in both bEnd.3 cells and vECs within isolated microvessels, supporting a direct effect of Aβ1‐40 on endothelial ferroptotic responses. Beyond the direct effects of Aβ, the intrinsic characteristics of the venous system may further contribute to vEC vulnerability. Venules are the major sites of leukocyte adhesion and transendothelial migration into the brain under inflammatory conditions,^44^ and the recruited immune cells may exacerbate oxidative stress by releasing reactive oxygen species (ROS).^45^ Moreover, venous endothelial cells exhibit greater sensitivity to oxidative stress compared with arterial endothelial cells.^46^ Given that ferroptosis is driven by iron‐dependent lipid peroxidation under conditions of insufficient antioxidant defense,^47^ Aβ‐induced oxidative stress, immune‐mediated inflammatory insults, and intrinsic redox vulnerability may collectively predispose cortical vECs to ferroptosis in CAA.

Understanding the molecular mechanisms underlying vEC ferroptosis in CAA is essential for uncovering potential therapeutic targets. Among the differentially expressed ferroptosis‐related genes, ATF3 emerged as a candidate regulator. Elevated ATF3 expression has previously been reported in age‐related neurodegenerative diseases and experimental models of cognitive decline.^48^, ^49^ In our study, ATF3 expression was increased in APP23 mice, with a similar trend observed in CAA patients, implicating ATF3 in CAA pathogenesis. ATF3 is a stress‐responsive transcription factor that is activated in response to diverse cellular insults, including Aβ exposure.^50^, ^51^ Previous studies have shown that ATF3 binds to the *SLC7A11* promoter and suppresses xCT expression,^40^ thereby influencing ferroptosis susceptibility in various pathologies.^52^, ^53^ Our ChIP‐qPCR analysis showed that Aβ1‐40 stimulation increased ATF3 enrichment at the predicted *Slc7a11* promoter region, whereas ATF3 knockdown restored xCT expression, supporting the involvement of ATF3 in *Slc7a11* transcriptional control. Aβ1‐40–induced ATF3 upregulation was also accompanied by reduced GPX4 expression and GSH levels, indicating compromised antioxidant defense and increased ferroptotic stress. Together, these findings support the involvement of the ATF3/xCT axis in vEC ferroptosis during CAA.

In vivo, endothelial ATF3 knockdown alleviated ferroptosis‐related alterations in vECs and reduced cerebral Aβ burden in APP23 mice. Previous studies reported increased ATF3 expression alongside cerebral Aβ accumulation in APP/PS1 mice,^54^ and more recent evidence suggests that ATF3‐mediated DOCK8 upregulation may promote Aβ plaque formation.^55^ Against this background, our intervention data suggest that endothelial ATF3 inhibition may also influence cerebral amyloid load. Given the contribution of the venous system to Aβ clearance, attenuation of ferroptotic injury by ATF3 inhibition may preserve venular endothelial function and thereby partly account for the reduced Aβ burden. Beyond ATF3/xCT‐mediated impairment of cystine uptake and GSH synthesis, the ferroportin downregulation observed in our study may provide an additional mechanism by promoting iron retention.

We also found that ATF3 knockdown in cerebrovascular endothelial cells ameliorated cognitive deficits in APP23 mice. Previous studies have implicated ATF3 in cognitive dysfunction; for example, Gao et al. reported that non–cell‐specific ATF3 silencing alleviated cognitive impairment in a rat model of vascular dementia by reducing oxidative stress and inflammation.^48^ Our findings extend these observations by suggesting a role for endothelial ATF3 in CAA‐associated cognitive dysfunction. The cognitive improvement following ATF3 knockdown in cerebrovascular endothelial cells likely involves multiple mechanisms. Reduced ferroptotic injury in vECs could help preserve venular endothelial function, while the concomitant decrease in cerebral Aβ burden may improve the neuronal microenvironment. ATF3 is also known to regulate oxidative stress and inflammatory responses,^48^, ^56^ and modulation of these pathways, together with broader attenuation of ferroptotic injury in cerebrovascular cells, could help preserve blood–brain barrier (BBB) integrity and maintain neurovascular homeostasis. These effects may collectively contribute to the observed improvement in cognitive function.

This study has several limitations that are important to note. First, although AAV‐BI30‐shATF3 effectively reduced ATF3 expression in vECs, this vector broadly targets cerebral endothelial cells rather than selectively targeting venous endothelium.^30^ Therefore, the observed therapeutic effects cannot be attributed exclusively to vECs. Future development of venule‐specific delivery strategies will be necessary to more precisely define the contribution of vEC ferroptosis to CAA progression. Second, our study primarily focused on early‐stage CAA using 11‐month‐old APP23 mice, in which hemorrhagic lesions, a feature of advanced CAA, have not yet developed.^57^ Whether early vEC ferroptosis contributes to subsequent hemorrhagic lesions remains to be determined in longitudinal studies and models of advanced disease. Third, ATF3 inhibition reduced both vEC ferroptosis‐related alterations and cerebral Aβ burden. However, the mechanisms linking vEC ferroptosis to Aβ clearance remain incompletely understood. In particular, how ferroptosis affects venular function and whether this influences cerebral waste clearance warrant further investigation, as methods for selectively assessing venular clearance function remain limited. Finally, although our preliminary observations in human CAA tissue suggested ferroptosis‐related alterations in cortical venules, the clinical relevance of the ATF3/xCT axis in vEC ferroptosis requires further validation. Due to the insidious onset and diagnostic challenges of early‐stage CAA,^58^ access to well‐characterized human vascular specimens remains limited. Given these challenges, future studies could use available human CAA brain tissue for single‐nucleus transcriptomic and spatially resolved histopathological analyses. Combining these tissue‐based findings with multimodal neuroimaging and blood‐ or cerebrospinal fluid‐based biomarkers may help determine whether ferroptotic alterations associated with the ATF3/xCT axis are present in human venules and assess their associations with disease severity, vascular dysfunction, and CAA‐related imaging features.

In summary, our study demonstrates that ferroptosis occurs in vECs during the early stage of CAA and that Aβ1‐40 induces this process via the ATF3/xCT axis. Importantly, ATF3 knockdown in cerebral endothelial cells, including vECs, reduces Aβ deposition and improves cognitive function. These findings support a pathogenic role of venous injury in CAA and highlight vEC ferroptosis as a novel and promising therapeutic target for the disease.

## CONFLICT OF INTEREST STATEMENT

The authors declare no conflicts of interest. Author disclosures are available in the Supporting Information.

## CONSENT STATEMENT

All CAA and non‐CAA human samples were provided by the National Human Brain Bank for Development and Function, Chinese Academy of Medical Sciences and Peking Union Medical College, Beijing, China. All human tissue samples were obtained with informed consent.

## Supporting information



Supporting information

Supporting information
