# Supplementary material for: Aβ1‐40 induces venular endothelial cell ferroptosis in cerebral amyloid angiopathy via the ATF3/xCT axis: Insights from single‐cell RNA sequencing
Source: Alzheimers Dement. 2026 Sep 20;22(9):e71858. doi: 10.1002/alz.71858 (PMC13590932; doi:10.1002/alz.71858)
Supplement: Supplementary file 2 [file ALZ-22-e71858-s002.docx]

**Part 1. Supplementary figures**

**Figure S1. Determination of the genotypes of APP23 mice.**

(**A**) Genotyping results of APP23 transgenic mice. PCR analysis demonstrates a specific band at approximately 250 bp was detected in APP23 mice, whereas no corresponding band was observed in WT mice.

(**B**) Western blot analysis of APP expression. Western blot analysis depicts APP protein was expressed in brain tissues of APP23 mice but not in WT mice.

(**C**) Representative immunofluorescence images showing Aβ deposition (6E10, green) around cerebral microvessels (CD31, red) in APP23 mice. Scale bar = 50um.

Abbreviations: NTC, no-template control; WT, wild type.

**Figure S2. Single-cell transcriptomic profiling of cerebral microvessels in APP23 mice.**

(**A**) UMAP visualization of all cells. Cells are color-coded by (left) sample, (middle) experimental group (APP23 vs. WT), and (right) seurat cluster assignment.

(**B**) Expression levels of canonical marker genes used to define the major cell types.

(**C**) Proportion of each major cell type across samples. Dot size represents the number of cells.

**Figure S3. Total iron content in the cortical tissue of APP23 and WT mice.**

(**A**) Representative images of iron deposition in cortical tissue of APP23 and WT mice at indicated ages. Iron was visualized by DAB-enhanced Perls’ Prussian blue staining, which appears as a brown precipitate. Scale bar = 200 µm.

(**B**) Box plots of relative Fe²⁺, Fe³⁺, and total iron levels in cortical tissue of 11- (upper panel) and 15-month-old (lower panel) mice. N = 5 per group.

Data are expressed as mean ± SD. ns, not significant; *P < 0.05; unpaired Student’s t-test (B).

**Figure S4. Representative images showing Aβ deposition in cerebral arteries of CAA patients.**

(**A**) Representative image of Congo red staining of leptomeningeal arteries from a brain section of the same CAA patient as in Figure 4N. Aβ is shown in red and nuclei in blue. Scale bar = 100 μm.

(**B**) Representative image of immunohistochemistry for MCT1 (brown) combined with Congo red (red) staining in a leptomeningeal artery (same section as in Figure 5A, top panel). Scale bar = 25 μm.

(**C**) Representative image of immunofluorescence co-staining for CD31 (green), α-SMA (red), and 6E10 (magenta) in a cortical artery (same section as in Figure 4O, Figure 5A, Figure 6D, bottom panel). Scale bar = 10 μm.

**Figure S5. Dose- and time-dependent effects of Aβ1–40 stimulation on ferroptosis-related molecules in bEnd.3 cells.**

**(A)** Representative Western blot analysis showing the protein expression levels of ACSL4, xCT, and GPX4 in bEnd.3 cells treated with different concentrations of oligomeric Aβ1–40 (0, 0.5, 1, 2, and 5 μM) for 12 h.

**(B)** Representative Western blot analysis showing the protein expression levels of ACSL4, xCT, and GPX4 in bEnd.3 cells treated with 1 μM oligomeric Aβ1–40 for different durations (0, 6, 12, and 24 h).

**(C)** Representative Western blot analysis showing the protein expression levels of ACSL4, xCT, and GPX4 in cortical microvessels treated with 1 μM oligomeric Aβ1–40 for different durations (0, 3, and 6 h).

**Figure S6. In-vitro experiment of lentivirus transduction in bEnd.3 cells.**

(**A**) Representative Western blot analysis demonstrating ATF3 protein levels following transduction with lentiviruses encoding three distinct ATF3-specific shRNAs (designated 1, 2, 3) or a NC shRNA.  Transductions were performed at different multiplicities of infection (MOI = 2, 10, 50) to assess knockdown efficiency. shRNA-2 at MOI=50 yielded the most potent and consistent ATF3 knockdown, and was therefore selected for subsequent experiments.

(**B**) Real-time quantitative PCR analysis of relative mRNA levels of *ATF3* and *Slc7a11* in various groups with lentivirus treatment. N = 6 per group.

Data are expressed as mean ± SD. *P < 0.05; ****P < 0.0001; ns, not significant; one-way ANOVA followed by Tukey's multiple comparisons test (B).

**Figure S7. In-vivo experiment of AAV-BI30 transduction in endothelial cells.**

Representative immunofluorescence images of cerebral endothelial cell-targeted AAV-BI30 infection in the WT and APP23 mice. Virus shows mCherry fluorescence (red), and endothelial cells are stained with CD31 (green).

**Part 2. Supplementary tables**

**Table S1. The demographic and clinical characteristics of the patients and controls.**

| No. | Group | ID | Sex | Age  (y) | Cause of death | Post-mortem delay (h) | Sample attributes | Sample site |
| --- | --- | --- | --- | --- | --- | --- | --- | --- |
| 1 | CAA | PTB637 | M | 90 | Pulmonary infection | 6.33 | Paraffin section | Visual cortex |
| 2 | CAA | PTB661 | M | 80 | Respiratory failure | 3.00 | Paraffin section | Visual cortex |
| 3 | CAA | PTB680 | F | 88 | Organ failure | 2.85 | Paraffin section | Visual cortex |
| 4 | Autopsy control | PTB742 | F | 85 | Pulmonary infection | 3.98 | Paraffin section | Visual cortex |
| 5 | Autopsy control | PTB750 | M | 80 | Respiratory failure | 4.23 | Paraffin section | Visual cortex |

**Table S2. Primers used in this study.**

| **Name** | **Sequence** | **Supplier** |
| --- | --- | --- |
| *Slc7a11-F* | 5’-TCATCATCGGCACCGTCATC-3’ | Servicebio |
| *Slc7a11-R* | 5’-TTACGAGCAGTTCCACCCAGAC-3’ | Servicebio |
| *Atf3-F* | 5’-TCGCCATCCAGAATAAACACC-3’ | Servicebio |
| *Atf3-R* | 5’-GCATTCACACTCTCCAGTTTCTCT-3’ | Servicebio |
| *β-actin-F* | 5’-AATCGTGCGTGACATCAA-3’ | Sangon Biotech |
| *β-actin-R* | 5’-GCTCGTTGCCAATAGTGA-3’ | Sangon Biotech |
| *Slc7a11-F* (chip-qpcr/pcr) | 5’-TCCATTGAGCAACCCACAGG-3’ | Sangon Biotech |
| *Slc7a11-R* (chip-qpcr/pcr) | 5’-ACCAACTTACTGGGCTGCAT-3’ | Sangon Biotech |

**Table S3. Lentivirus and AAV.**

| **Name** | **Sequence** **(5′–3′)** | **Supplier** |
| --- | --- | --- |
| Lentivirus (pLKO.1-mCherry-P2A-Puro-shATF3) | | |
| shRNA1 | GCTAACCTGACACCCTTTGTC | Paiwei Biotechnology |
| shRNA2 | GACACCCTTTGTCAAGGAAGA | Paiwei Biotechnology |
| shRNA3 | GCTGAGATTCGCCATCCAGAA | Paiwei Biotechnology |
| shNC | / | Paiwei Biotechnology |
| AAV-BI30 | | |
| pAAV-TIE1p-MCS-mCherry-miR30shRNA (ATF3)-WPRE | GACACCCTTTGTCAAGGAAGA | OBiO Technology |
| pAAV-TIE1p-MCS-mcherry-miR30shRNA (NC)-WPRE | GAAGTCGTGAGAAGTAGAA | OBiO Technology |

**Table S4. Key Resources.**

| **Reagents** | **Source** | **Identifier** |
| --- | --- | --- |
| **Antibodies** |  |  |
| β-actin | Servicebio | GB15003-100 |
| 6E10 | BioLegend | 803001 |
| FTH (Ferritin Heavy Chain) | Santa Cruz Biotechnology | sc-376594 |
| FTL (Ferritin Light Chain) | Proteintech | 10727-1-AP |
| Ferritin | Abcam | ab75973 |
| Ferroportin (SLC40A1) | Novus Biologicals | NBP1-21502 |
| TFRC (CD71, Transferrin Receptor) | Santa Cruz Biotechnology | sc-65882 |
| DMT1 (SLC11A2) | Proteintech | 20507-1-AP |
| GPX4 (Glutathione Peroxidase 4) | Abcam | ab125066 |
| xCT (SLC7A11) | Abclonal | A2413 |
| ACSL4 (Acyl-CoA Synthetase Long Chain Family Member 4) | Proteintech | 22401-1-AP |
| ATF3 (Activating Transcription Factor 3) | Abcam | EPR19488 |
| CD31 (PECAM-1) | BD Pharmingen | 550274 |
| MCT1 (SLC16A1) | Proteintech | 20139-1-AP |
| α-SMA (Alpha-Smooth Muscle Actin) | Cell Signaling Technology | 19245S |
| α-SMA (Alpha-Smooth Muscle Actin) | Santa cruz biotechnology | sc-53142 |
| Goat Anti-Rabbit IgG, HRP | Servicebio | GB23303 |
| Goat Anti-Mouse IgG, HRP | Servicebio | GB23301 |
| Donkey anti-Rabbit IgG (H+L), Alexa Fluor™ 647 | Yeasen | 34213ES60 |
| Donkey anti-Mouse IgG (H+L), Alexa Fluor™ 488 | Abcam | ab150105 |
| Donkey anti-Rat IgG (H+L), Alexa Fluor™ 594 | Abcam | ab150156 |
| Donkey anti-Rabbit IgG (H+L), Alexa Fluor™ 488 | Yeasen | 34206ES60 |
| Donkey anti-Mouse IgG (H+L), Alexa Fluor™ 488 | Yeasen | 34106ES60 |
| **Chemicals** |  |  |
| Aβ1-40 | Chinesepeptide | AMYD-009 |
| Liproxstatin-1 | Merck | SML1414 |
| **Critical commercial assays** | **Source** | **Identifier** |
| Iron Assay Kit | Abcam | ab83366 |
| GSH and GSSG Assay Kit | Beyotime | S0053 |
| Lipid Peroxidation MDA Assay Kit | Beyotime | S0131S |
| Cell Counting Kit-8 (CCK8) | Servicebio | G4103 |
| AEC Substrate Kit | MXB | AEC-0037 |
| DAB Substrate Kit | MXB | DAB-4033 |
| Enhanced Prussian Blue Iron Stain Kit | Solarbio | G1428 |
| Congo Red Amyloid Stain Kit (Bennhold Method) | Solarbio | G1530 |
| SteadyPure RNA Extraction Kit | Accurate Biology | AG21024 |
| PrimeScript RT Master Mix | Takara | RR036A |
| SYBR Green Premix Pro Taq HS qPCR Kit | Accurate Biology | AG11701 |
| SeekOne ® DD Single Cell 3’ Transcriptome-seq Kit | SeekGene | K00202 |
| VAHTS DNA Clean Beads | Vazyme | N411-01 |
| One Step Mouse Genotyping Kit | Vazyme | PD101-01 |
| ChIP Assay Kit | Beyotime | P2078 |
| FastPure Gel DNA Extraction Mini Kit | Vazyme | DC301 |
| **Culture medium and Supplement** |  |  |
| Dulbecco's Modified Eagle Medium (High glucose) | Pricella | PM150210 |
| Fetal bovine serum | Servicebio | G8003 |
| Penicillin/streptomycin | Servicebio | G4003 |
